# FBXO28 suppresses liver cancer invasion and metastasis by promoting PKA-dependent SNAI2 degradation

**DOI:** 10.1038/s41388-023-02809-0

**Published:** 2023-08-18

**Authors:** Xinran Qiao, Jingyu Lin, Jiajia Shen, Yang Chen, Liyun Zheng, Hangjiang Ren, Xiaoli Zhao, Hang Yang, Pengyu Li, Zhen Wang

**Affiliations:** 1 Institute of Medicinal Biotechnology, Chinese Academy of Medical Sciences and Peking Union Medical College, Beijing, China; 2 The Affiliated Cancer Hospital of Zhengzhou University & Henan Cancer Hospital, Zhengzhou, Henan Province China; 3 Qilu Hospital of Shan Dong University, Jinan, Shandong Province China

**Keywords:** Liver cancer, Metastasis, Ubiquitylation, Tumour biomarkers

## Abstract

FBXO28 is a member of F-box proteins that are the substrate receptors of SCF (SKP1, CULLIN1, F-box protein) ubiquitin ligase complexes. Despite the implications of its role in cancer, the function of FBXO28 in epithelial-mesenchymal transition (EMT) process and metastasis for cancer remains largely unknown. Here, we report that FBXO28 is a critical negative regulator of migration, invasion and metastasis in human hepatocellular carcinoma (HCC) in vitro and in vivo. *FBXO28* expression is upregulated in human epithelial cancer cell lines relative to mesenchymal counterparts. Mechanistically, by directly binding to SNAI2, FBXO28 functions as an E3 ubiquitin ligase that targets the substrate for degradation via ubiquitin proteasome system. Importantly, we establish a cooperative function for PKA in FBXO28-mediated SNAI2 degradation. In clinical HCC specimens, FBXO28 protein levels positively whereas negatively correlate with PKAα and SNAI2 levels, respectively. Low *FBXO28* or *PRKACA* expression is associated with poor prognosis of HCC patients. Together, these findings elucidate the novel function of FBXO28 as a critical inhibitor of EMT and metastasis in cancer and provide a mechanistic rationale for its candidacy as a new prognostic marker and/or therapeutic target in human aggressive HCC.

## Introduction

Hepatocellular carcinoma (HCC) accounts for over 75% of all liver cancers and is a significant cause of cancer-related mortality worldwide owing to resistance to chemotherapy and frequent recurrence and metastasis, which leads to poor prognosis with a 5-year survival rate of 15–38% [1]. Therefore, addressing the mechanisms underlying HCC progression and metastasis will help develop new treatment strategy.

Epithelial-mesenchymal transition (EMT) is a reversible process that contributes to cancer metastasis, invasion and chemoresistance as well as promotes the generation of cancer stem cells [2]. The best-characterized core EMT regulatory factors are the members of the Snail family SNAI1 and SNAI2 (formerly known as Snail and Slug, respectively), both of which have been known as zinc finger transcriptional repressors to regulate the expression of E-cadherin, a key epithelial marker, as well as other genes involved in cell adhesion [3, 4]. Elevated expression of SNAI1 and SNAI2 have been observed in a variety of cancer types and correlate with increased risks of metastasis and postoperative relapse, predicting poor prognosis for the survival in cancer patients [5]. The Snail family members are short-lived proteins, the stability of which is normally controlled by the ubiquitin proteasome system (UPS) [6]. Relative to SNAI1, regulatory machinery controlling the homeostasis levels of SNAI2 protein under EMT contexts in cancer especially HCC, is less known.

F-box proteins are the substrate receptors of SKP1- CULLIN1-F-box protein (SCF) ubiquitin ligase complexes that contain 69 family members in humans. Recent studies have shed light on the regulatory functions of several F-box proteins during the EMT process by either directly participating in the degradation of EMT transcriptional factors, or indirectly controlling EMT signaling inducers [7, 8]. Despite these studies, the potential functions for other F-box proteins in regulating EMT and cancer metastasis still remains to be determined.

FBXO28 is an evolutionary conserved F-box protein that was initially identified as an interactor of DNA Fragmentation Factor 45 (DFF45) in a yeast two/three hybrid screening [9]. Several studies have suggested a significant contribution for FBXO28 to the neurodevelopmental defects in the 1q41q42 microdeletion syndrome [10]. FBXO28 also ensures proper mitotic progression via interacting with Type IIα topoisomerases and regulating its decatenation activity [11]. Moreover, the SCF^FBXO28^ protein complex has been identified as an E3 ubiquitin ligase that promotes MYC-driven transcription and tumorigenesis [12], while inhibits the survival of glioblastoma via interfering with PFKFB4 and promoting the degradation of HIF-1α [13]. FBXO28 is also found to be upregulated in breast cancer (BC) and pancreatic ductal adenocarcinoma (PDAC), and high FBXO28 expression might serve as a poor prognostic factor for BC and PDAC [14, 15]. In addition, FBXO28 reportedly has a complicated correlation with the survival and treatment outcome in stratified BC patients [16]. Up to now, the function of FBXO28 in HCC related EMT process and metastasis remains completely unknown.

In the present study, we demonstrate that FBXO28 negatively regulates EMT progression and has a significant protection against migration, invasion and metastasis in HCC. Mechanistically, by binding to SNAI2, FBXO28 functions as an unidentified SCF ubiquitin ligase that specifically targets the substrate for degradation.

## Results

### FBXO28 inhibits migration, invasion and lung metastasis of HCC cells

**Fig. 1:**
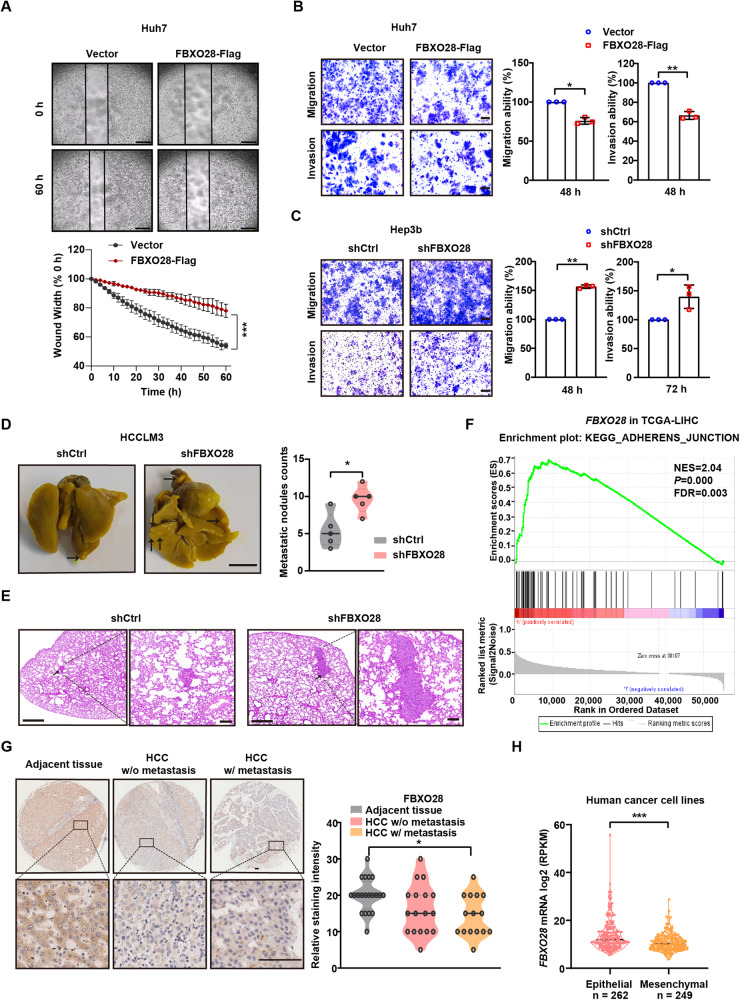
FBXO28 inhibits migration, invasion and metastasis of HCC cells. **A–C** Huh7 cells were transfected with pCMV6-FBXO28-Myc-Flag (FBXO28-Flag) or a vector control for 48 h, or Hep3b cells were infected with lentivirus expressing shFBXO28 or shCtrl and stably selected, followed by wound healing (**A**) as well as migration and invasion assays (**B**, **C**). Representative images are shown in (**A**) (Scale bar: 0.5 mm) and (**B**, **C**) (Scale bar: 10 μm). Data in (**A**–**C**) were presented as mean ± SD from three independent experiments. *P < 0.05, **P < 0.01 and ***P < 0.001 (independent *T*-test). **D** HCCLM3 cells (2 × 10^6^) stably expressing shFBXO28 or shCtrl were injected into NOD/SCID mice via tail veins, and the number of lung metastatic nodules was counted after the mice were sacrificed at 10 weeks after injection (*n* = 5). Scale bar: 4 mm, *P < 0.05 (independent *T*-test). **E** Hematoxylin and eosin staining of the indicated lung metastatic nodules. The scale bars in left and right panel represent 500 μm and 100 μm, respectively. **F** GSEA enrichment plot from HCC cohorts. **G** Immunochemistry staining of HCC tissue microarray. Representative sample from each group (normal, primary, and metastatic) was shown on the left panel. Scale bar: 100 μm. The staining intensity was quantified on the right panel. *P < 0.05 (one-way ANOVA). **H** *FBXO28* mRNA levels in epithelial and mesenchymal cancer cell lines plotted based on the RPKM. ***P < 0.001 (independent *T*-test).

We selected several F-box proteins (FBXO15, FBXO17, FBXO24, FBXO28, FBXO46, FBXW8, FBXL7) whose functions are scarcely reported in EMT and metastasis of HCC, and evaluated their effect on the migration capability of Huh7 cells in a transwell assay upon gene knockdown. Among the seven genes, *FBXO28* knockdown resulted in a marked increase in the migration capability at both 24 and 48 h compared with the control group (Supplementary Fig. S1A, B), indicating that FBXO28 might inhibit the migration of HCC. By overexpressing FBXO28 in Huh7 cells having higher metastatic trait or knocking down FBXO28 expression in Hep3b cells with relatively lower metastatic capability, we tested whether FBXO28 affects cell migration and invasion capability. Efficiency of FBXO28 overexpression and knockdown in the HCC cells were shown in Supplementary Fig. S1C, D. As observed in Fig. 1A–C and Supplementary Fig. S1E, FBXO28 overexpression in Huh7 cells markedly suppressed, whereas FBXO28 knockdown in Hep3b cells greatly promoted, the cell migration and invasion capability within 48 or 72 h by wound-healing and transwell assays. We also analyzed the cell proliferation and viability by counting number and ATPlite assay, respectively, under the same serum-free conditions as in migration process, and no significant changes for cell number and viability were observed (Supplementary Fig. S1F–H), thus ruling out the possibility that cell number changes might affect migrative process.

To confirm above findings, we further performed a rescuing experiment by re-expressing FBXO28 in Hep3b and Huh7 cells depleted of FBXO28 by RNAi-mediated knockdown or CRISPR-mediated knockout, respectively. As seen in the Supplementary Fig. S2A–E, FBXO28 deficiency consistently enhanced cell migration ability, while re-expression of the protein markedly reduced the potentiated migration ability in either cell line, fully supporting a negative regulation of FBXO28 toward HCC migration.

Moreover, we noticed a significant increase in the metastatic pulmonary nodules in FBXO28-depleted HCCLM3 cells relative to the control group in mice model (Fig. 1D, E, Supplementary Fig. S1D). Furthermore, GSEA enrichment analysis showed that high *FBXO28* expression is positively correlated with the enrichment of the adherens junction signaling pathway in HCC cohorts from The Cancer Genome Atlas (TCGA) dataset (Fig. 1F). In addition, IHC analysis of tissue array containing thirty-two HCC specimens including several metastatic samples showed a decreased tendency of FBXO28 staining in HCC without metastasis compared with that in adjacent tissues, with more significantly downregulated FBXO28 expression observed in metastatic specimens (Fig. 1G). Supportively, a decreased expression of *FBXO28* in mesenchymal cancer cell lines relative to epithelial counterparts was observed via transcriptome analysis of a large set of cancer cells (Fig. 1H) [17]. Altogether, these data strongly support an inhibitory role of FBXO28 in migratory, invasive and lung metastatic process of HCC cells.

### FBXO28 inhibits HCC cellular proliferation in vitro while has negligible tumor-suppressing effect in vivo

**Fig. 2:**
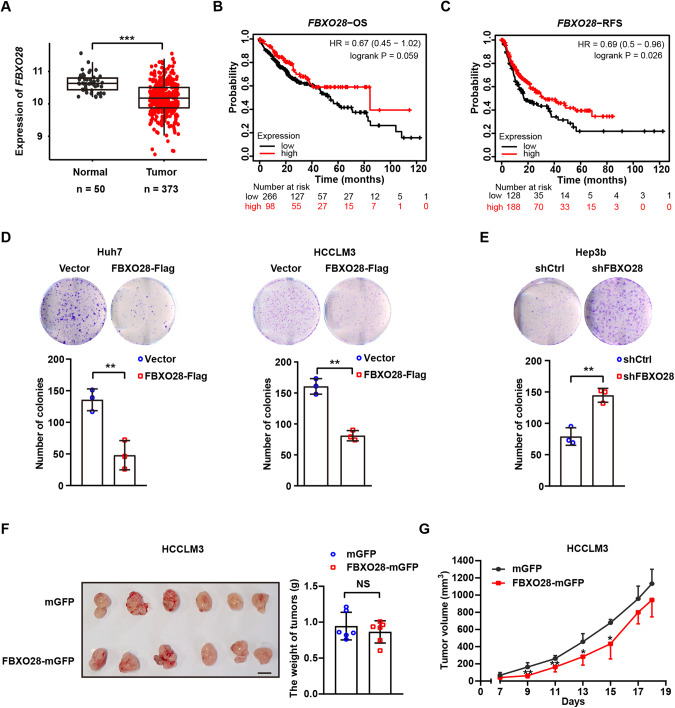
Effects of FBXO28 on HCC survival and proliferation in vitro and in vivo. **A** *FBXO28* mRNA levels in HCC and normal cohorts in TCGA. ***P < 0.001 (independent *T*-test). **B**, **C** Kaplan–Meier survival analysis of liver cancer patients. Correlation of *FBXO28* expression and overall survival (OS) and relapse-free survival (RFS) were shown in (**B**) and (**C**), respectively. **D**, **E** Huh7 and HCCLM3 cells stably expressing FBXO28-Flag or a vector control (**D**) or Hep3b cells stably expressing shFBXO28 or shCtrl (**E**) were plated in 6-well plates for 14 days for colony formation assay. Representative images are shown. Data were presented as mean ± SD from three independent experiments. **P < 0.01 (independent *T*-test). **F**, **G** HCCLM3 cells stably expressing FBXO28-mGFP or mGFP control vector were inoculated into BALB/c nude mice. The length and width of the tumor masses were measured every 2 days as described in *Materials and Methods*. Average tumor weight on day 18th after inoculation (**F**) and tumor volume (**G**) during the indicated time were calculated (mean ± SD; *n* = 6 per group). Scale bar: 10 mm. *P < 0.05, **P < 0.01 versus the mGFP group at indicated time (independent *T*-test). NS no significance.

Next, we analyzed the expression pattern in HCC cohorts from TCGA and found a marked underexpression of *FBXO28* gene in cancerous tissue relative to normal counterparts (Fig. 2A). Patients with higher FBXO28 expression exhibit longer overall survival (OS) and relapse-free survival (RFS) time than those with lower expression via Kaplan–Meier survival analysis in HCC cohorts (www.kmplot.com) (Fig. 2B, C). These results suggest that low expression of *FBXO28* is closely associated with aggressiveness for HCC patients and indicate poor prognosis.

As enhanced invasiveness for metastasis is normally associated with tumorigenesis in cancer, we next investigated whether FBXO28 has a regulatory role in HCC proliferation under normal culture conditions. As seen in Fig. 2D, E and Supplementary Fig. S1D, FBXO28 overexpression in Huh7 and HCCLM3 cells significantly suppressed whereas FBXO28 depletion in Hep3b cells greatly promoted cellular survival in the colony formation assay within 2 weeks. However, while the tumor volume in FBXO28-overexpressed HCCLM3 cell xenograft mice model showed a decreased trend relative to the control group during the observation time after inoculation, no significant changes were found for tumor weight and volume between the two groups on the 18th day (Fig. 2F, G, Supplementary Fig. S3A). Similar negligible effects were observed in FBXO28-depleted Hep3b cells xenograft model compared with control group (Supplementary Fig. S3B–D).

### FBXO28 negatively regulates the turnover and function of SNAI2

**Fig. 3:**
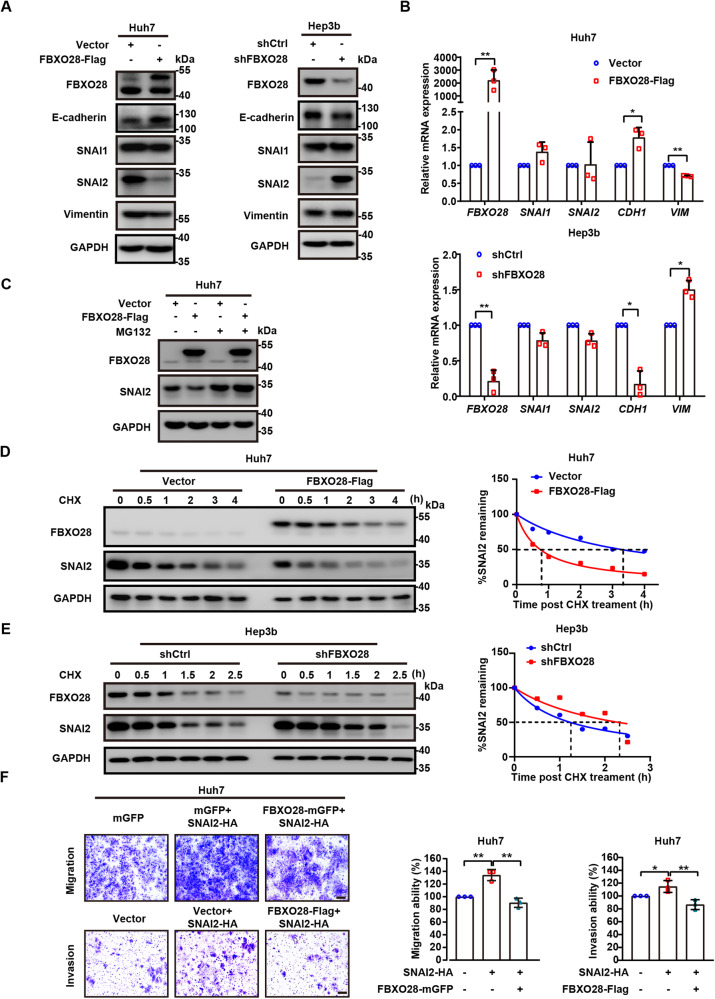
FBXO28 negatively regulates SNAI2 protein levels and function. **A** Huh7 cells transfected with FBXO28-Flag or vector for 48 h (left panel) or Hep3b infected with lentivirus expressing shFBXO28 or shCtrl for 72 h (right panel) were subjected to IB analyses of the EMT pathway-related markers. **B** Total cellular RNA was extracted for RT-qPCR analysis in the same conditions as in (**A**). Data were presented as mean ± SD from three independent experiments, *P < 0.05 and **P < 0.01 (independent *T*-test). **C** Huh7 cells expressing FBXO28-Flag or vector for 72 h by transient transfection were treated with MG132 for 6 h prior to IB analyses. **D**, **E** Huh7 cells transfected with FBXO28-Flag or a control vector (**D**) or Hep3b cells expressing shFBXO28 or shCtrl for 48 h (**E**) were treated with CHX (10 μg/mL) for the indicated time interval and subjected to IB analyses. The SNAI2 protein levels in (**D** and **E**) were quantified, respectively, and shown on the right panel. **F** Huh7 cells stably expressing FBXO28-mGFP or a control vector were transfected with SNAI2-HA for 48 h; or were transfected with SNAI2-HA or a control vector for 24 h prior to transfection with FBXO28-Flag for 24 h, followed by migration and invasion assay, respectively. Representative images are shown. Scale bar: 10 μm. Data were expressed as mean ± SD from three independent experiments. *P < 0.05 and **P < 0.01 (one-way ANOVA).

Based on above findings, we reasoned that FBXO28 may play a pivotal role in affecting the expression of EMT regulatory factors. Indeed, overexpression of FBXO28 in Huh7 cells led to a significant decrease in the protein levels of SNAI2 and mesenchymal marker Vimentin, as well as an increase in the levels of epithelial marker E-cadherin, whereas silencing of FBXO28 in Hep3b cells demonstrated an opposite effect (Fig. 3A). Notably, less significant change of SNAI1 was observed relative to SNAI2 levels under the same conditions (Fig. 3A, Supplementary Fig. S4A). Further qRT-PCR analysis showed that the transcriptional levels of *SNAI1* and *SNAI2* remained unaffected while the expression of *CDH1* and *VIM* was similarly regulated as the pattern of respective proteins upon altering FBXO28 expression (Fig. 3B). These results indicate that FBXO28 may regulate the SNAI2 protein at a post-transcriptional level. Supportively, the increased SNAI2 expression upon FBXO28 depletion in Hep3b cells was markedly reduced by FBXO28 re-expression (Supplementary Fig. S4B); also increasing expression of FBXO28 in HCCLM3 cells led to a gradual reduction of endogenous SNAI2 protein levels (Supplementary Fig. S4C). Moreover, the FBXO28 inhibited SNAI2 protein level was fully rescued by treatment with a proteasome inhibitor MG132 in the Huh7 cells as well as human breast cancer MDA-MB-231, lung cancer NCI-H460 and osteosarcoma Saos-2 cell lines (Fig. 3C; Supplementary Fig. S4D). Consistently, a shortened and extended half-life for SNAI2 was observed in FBXO28-overexpressed Huh7 and FBXO28-depleted Hep3b cells, respectively, after treatment with cycloheximide (CHX), a protein synthesis inhibitor (Fig. 3D, E). These data indicate that FBXO28 may reduce the physiological SNAI2 protein levels via UPS, independent of tissue type. Supportively, FBXO28 up-regulation markedly suppressed the migration and invasion capability induced by ectopic SNAI2 expression in Huh7 cells (Fig. 3F), confirming FBXO28 inhibits SNAI2’s function. Given that MYC is a target of FBXO28 in HCT116 cells [12] and inhibits metastasis as well [18], we further investigated whether MYC may be affected and detected no change of MYC protein levels by FBXO28 overexpression or knockdown in HCC cells (Supplementary Fig. S4E), excluding the possible involvement of MYC under this context. Collectively, our data suggest that FBXO28 negatively and specifically regulates the SNAI2 protein turnover and its function in HCC.

### FBXO28 interacts with SNAI2 and promotes the protein for proteasome-mediated degradation

**Fig. 4:**
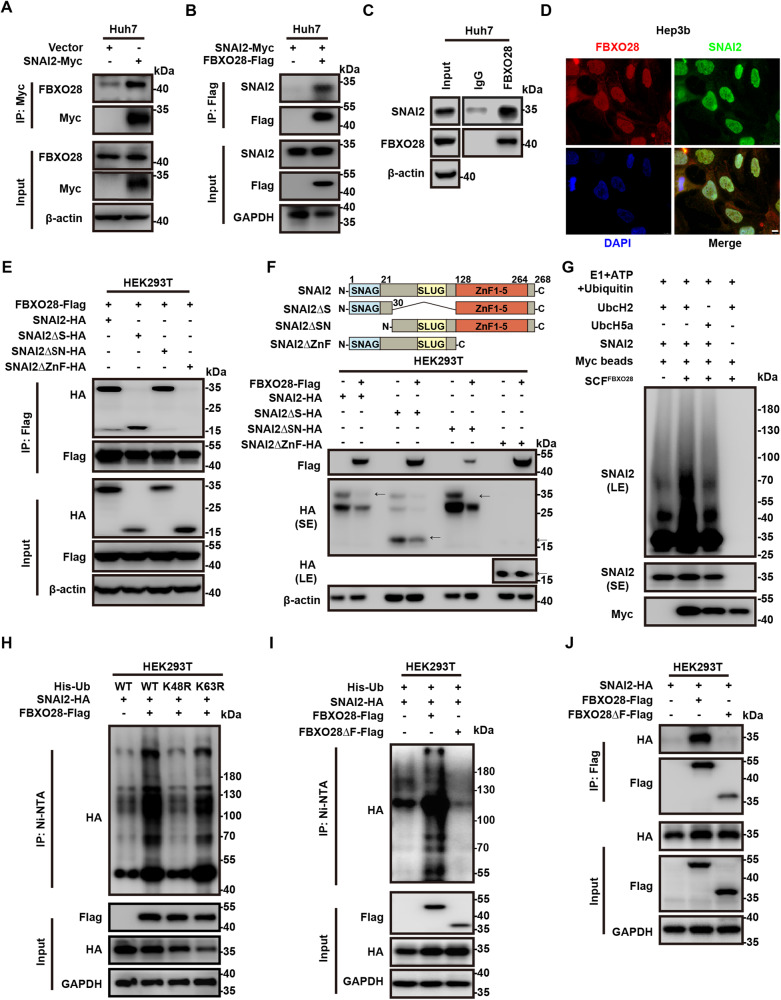
FBXO28 interacts with, and ubiquitylates, SNAI2. **A**, **B** Huh7 cells were transfected with indicated plasmids for 72 h, and then subjected to IP and IB analyses. **C** Huh7 cells were subjected to IP with FBXO28 antibody or normal rabbit IgG and IB analyses. **D** Co-localization of FBXO28 and SNAI2 by immunofluorescence staining in Hep3b cells. Representative images are shown. Scale bar: 8 μm. **E** HEK293T cells co-expressing FBXO28 and full-length or deletion mutants of SNAI2 (with schematic diagram shown on the upper panel in **F**) were cultured for 48 h prior to IP and IB analyses. **F** Upper panel: A schematic representation of SNAI2 deletion mutants. Lower panel: HEK293T cells transfected with indicated plasmids for 48 h were subjected to IB analyses. Arrows indicate the target band. **G** In vitro ubiquitination assay using cell immunocomplex pulled down with anti-Myc beads from HEK293T cell extracts after transfection with FBXO28-Flag plasmid for 48 h. The polyubiquitination was detected by IB with anti-SNAI2 antibody. **H** HEK293T cells were co-transfected with indicated plasmids for 72 h. The polyubiquitylated proteins were purified by Ni-NTA beads and detected with anti-HA antibody. **I** HEK293T cells were transfected with indicated plasmids for 72 h followed by Ni-NTA beads purification and IB with anti-HA antibody. **J** HEK293T cells were transfected with indicated plasmids for 48 h prior to IP and IB analyses. SE short exposure. LE long exposure.

Next, we investigated whether FBXO28 and SNAI2 have direct interaction. Exogenously expressed SNAI2-Myc in Huh7 cells was found to bind to endogenous FBXO28 by co-IP analysis (Fig. 4A). Meanwhile, overexpression of FBXO28-Flag was found to co-immunoprecipitate SNAI2-Myc in Huh7 and HCCLM3 cells by anti-Flag antibody (Fig. 4B and Supplementary Fig. S5A). The endogenous binding between FBXO28 and SNAI2 proteins was also validated by co-IP analysis with anti-FBXO28 antibody in Huh7 cells (Fig. 4C). IF analysis further proved FBXO28 and SNAI2 proteins were primarily co-localized in the nucleus (Fig. 4D). Also, co-IP analysis indicated that FBXO28 could form a complex with CULLIN1 and SKP1 in HEK293T cells, both of which belong to the subunit of SCF family complex, thus supporting FBXO28 functions as a member of SCF^FBXO28^ complex (Supplementary Fig. S5B).

We then mapped the potential region within SNAI2 protein that is responsible for its interaction with FBXO28. By generating several truncation mutations in which the SNAG (ΔSN), SLUG (ΔS) or Zinc finger (ΔZnF) domain of SNAI2 was removed respectively (as illustrated in Fig. 4F, upper panel), we found that the ΔZnF mutant construct failed to bind to FBXO28 (Fig. 4E), indicating the Zinc finger domain in C-terminal is critical for SNAI2’s associating with FBXO28. Supportively, the ΔZnF mutant construct remained insensitive whereas other mutant constructs were sensitive to FBXO28-induced degradation upon co-transfection in HEK293T cells (Fig. 4F, lower panel).

To investigate whether FBXO28 functions as an E3 ligase that ubiquitylates SNAI2, FBXO28-Flag, SNAI2-HA, and His-Ub expressing vectors were co-transfected into HEK293T cells for 72 h and MG132 was added 6 h before Ni-NTA purification. The polyubiquitinated SNAI2 was purified by Ni-NTA beads under denaturing conditions and detected by IB with anti-HA antibody. As shown in Fig. 4H, ectopic expression of FBXO28 strongly stimulated the polyubiquitination of SNAI2. We also found FBXO28 overexpression facilitated the K48-linked, instead of the K63-linked, polyubiquitination of SNAI2, as proved by the abrogation of polyubiquitinated SNAI2 upon co-transfection with mutant His-Ub K48R instead of His-Ub K63R (Fig. 4H). Given that the K48-linked polyubiquitination of target proteins normally leads to proteasome-mediated degradation, whereas K63-linked ubiquitination results in enhanced substrates stability and activity [19, 20], this result confirmed the proteolysis for the substrate via UPS. Conversely, the polyubiquitination of SNAI2 was decreased in FBXO28-depleted Hep3b cells pulled down by anti-HA magnetic beads in an in vivo ubiquitination assay (Supplementary Fig. S5C). Moreover, by screening a panel of E2 ubquitin-conjugation enzymes that may mediate SNAI2’s polyubiquitination, we chose UbcH2 as a functional E2 for the in vitro ubiquitylation assay (Supplementary Fig. S5D) and supportively, anti-Myc immunoprecipitated SCF^FBXO28^ complex promoted the polyubiquitination of SNAI2 protein when E1, ATP and Ubiquitin were concomitantly added along with UbcH2 instead of UbcH5a (Fig. 4G).

To further confirm the F-box domain, the sequence required to interact with the SCF complex [7], is pivotal for the polyubiquitination of SNAI2 by FBXO28, we used a mutant construct lacking the F-box domain (FBXO28ΔF). Expectedly, FBXO28ΔF failed to promote the polyubiquitination of SNAI2 analyzed by Ni-NTA beads purification (Fig. 4I). However, apart from the expectedly inhibition of binding between FBXO28ΔF and SKP1 in HEK293T cells (Supplementary Fig. S5E), we also observed a marked reduction of binding between SNAI2 and FBXO28ΔF compared to the wild type (WT) FBXO28 (Fig. 4J), indicating the F-box domain is pivotal for FBXO28’s association with both SNAI2 and SKP1. Consistently the F-box-substrate binding pattern is observed for other F-box proteins such as FBXO31 and FBXO45 [21, 22]. Another intriguing finding was that although the polyubiquitination of SNAI2 was inhibited by FBXO28ΔF, a significant suppression of SNAI2 protein levels was observed in Huh7 cells overexpressing the mutant construct (Supplementary Fig. S5F). This is possibly due to an unexpected downregulation of *SNAI2* mRNA by the mutant construct via a yet unknown mechanism (Supplementary Fig. S5G). Altogether, these data confirm FBXO28 is a bona fide E3 ligase that binds to SNAI2 and targets the substrate for degradation via UPS.

### PKA is required for FBXO28-mediated SNAI2 decay

**Fig. 5:**
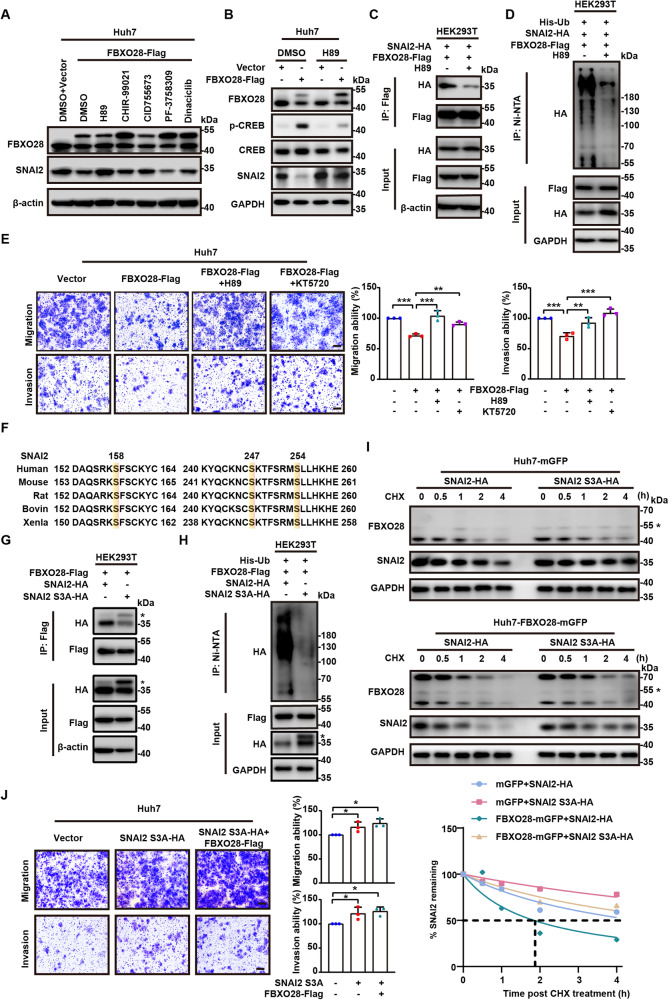
PKA-dependent phosphorylation is pivotal for FBXO28-mediated SNAI2 degradation. **A** Huh7 cells transiently expressing FBXO28-Flag were treated with DMSO or 0.2 μM H89, 4 μM CHIR-99021, 5 μM CID755673, 0.5 μM PF-3758309, 2 nM Dinaciclib for 24 h, followed by IB analyses. **B** Huh7 cells transiently expressing FBXO28-Flag or a control vector were treated with DMSO or 0.5 μM H89 for 24 h prior to IB analyses. **C** HEK293T cells were transfected with indicated plasmids for 24 h, and then treated with DMSO or 0.2 μM H89 for 24 h, followed by IP and IB analyses. **D** HEK293T cells were transfected with indicated plasmids for 48 h prior to treatment with DMSO or 0.2 μM H89 for 24 h. The polyubiquitylated proteins were purified by Ni-NTA beads and detected with anti-HA antibody. **E** Huh7 cells expressed FBXO28-Flag or a control vector for 24 h were treated in the absence or presence of 0.5 μM H89 or 1 μM KT5720 for another 24 h, followed by transwell assays. Scale bar: 10 μm. Data were presented as mean ± SD (*n* = 3). **P < 0.01 and ***P < 0.001 (one-way ANOVA). **F** Illustration of conservative amino acids sequences in the C-terminal of SNAI2 across different species using UniProt dataset. Predicted phosphorylation sites by PKA are highlighted. **G**, **H** HEK293T cells were transfected with indicated plasmids for 48 h and subjected to IP and IB analyses in (**G**) or Ni-NTA beads purification in (**H**). The asterisks represent non-specific band. **I** Huh7 cells stably expressing FBXO28-mGFP or a control vector were transfected with SNAI2-HA or SNAI2 S3A-HA for 48 h and treated with CHX for the indicated time interval, followed by IB analyses. **J** Huh7 cells were expressed with SNAI2 S3A-HA or a control vector for 24 h prior to transfection with FBXO28-Flag for another 24 h, followed by transwell assays. Scale bar: 10 μm. Data were shown as mean ± SD (*n* = 3). *P < 0.05 (one-way ANOVA).

F-box proteins are well known to generally recognize the phosphorylated protein substrates [7], and the Snail family have been reported to be either commonly or separately phosphorylated by a panel of protein kinases before being recognized by E3 ligases, which include glycogen synthase kinase 3 beta (GSK3β), protein kinase D1 (PKD1), protein kinase A (PKA), cyclin-dependent kinase 2 (CDK2) and p21 activated kinase 1 (PAK1) [23–27]. Thus, we used specific inhibitors of these kinases to assess whether the phosphorylation by a protein kinase may be critical in FBXO28-regulated SNAI2 decay. As shown in Fig. 5A, addition of the PKA inhibitor H89, instead of other kinase inhibitors including GSK3β inhibitor CHIR-99021, PKD1 inhibitor CID755673, CDK2 inhibitor Dinaciclib, PAK inhibitor PF-3758309, was found to be capable of fully rescuing the suppressed SNAI2 protein levels upon FBXO28 overexpression, suggesting a possible role of PKA kinase activity for FBXO28-regulated SNAI2 decay. Supportively, H89-restored SNAI2 protein under FBXO28 overexpression was accompanied by a significantly reduced PKA activity, as evidenced by the suppressed expression of PKA substrate p-CREB levels (Fig. 5B). Similar rescuing results were observed using another PKA inhibitor KT5720 as well as PKAα (a pivotal PKA subunit) knockdown (Supplementary Fig. S6A, E). We next hypothesized that PKA-dependent phosphorylation may be vital for the interaction between FBXO28 and SNAI2. Indeed, treatment with either H89 or KT5720 greatly inhibited the binding of both proteins by IP analysis (Fig. 5C, Supplementary Fig. S6B). Accordingly, the FBXO28-caused polyubiquitination of SNAI2 was markedly inhibited by either PKA inhibitor treatment assayed by Ni-NTA purification (Fig. 5D, Supplementary Fig. S6C). We further analyzed whether either PKA inhibitor may affect the biological functions. Expectedly, co-treatment with either H89 or KT5720 was capable of restoring the FBXO28-suppressed migration and invasion capability to a level comparable with control group in Huh7 cells (Fig. 5E). Interestingly, an extended half-life of endogenous SNAI2 levels by H89 treatment alone in Huh7 cells was also observed, supporting that PKA activation promotes the static SNAI2 turnover (Supplementary Fig. S6D).

Given that FBXO28 interacts with SNAI2 via its C-terminal zinc finger domain, we further assessed what putative residues in this domain could be specific PKA phosphorylation site. By analyzing the sequences using a kinase specific phosphorylation site prediction tool [28], residues at Ser158, Ser247 and Ser254 were predicted to be phosphorylated by PKA and recognized by FBXO28 (Supplementary Fig. S6G). These residues in SNAI2 are also highly conserved across different species (Fig. 5F). Compared with WT SNAI2, mutation of the three residues (S158A, S247A and S254A, abbreviated as SNAI2 S3A) greatly compromised the interaction of SNAI2 with FBXO28, resulting in the reduction of its polyubiquitination level (Fig. 5G, H). Moreover, the protein half-life of SNAI2 S3A was markedly extended relative to WT SNAI2, and remained unaffected by the overexpressed FBXO28 (Fig. 5I). Similarly, FBXO28 failed to affect the migration and invasion capability in Huh7 cells overexpressing SNAI2 S3A (Fig. 5J).

**Fig. 6:**
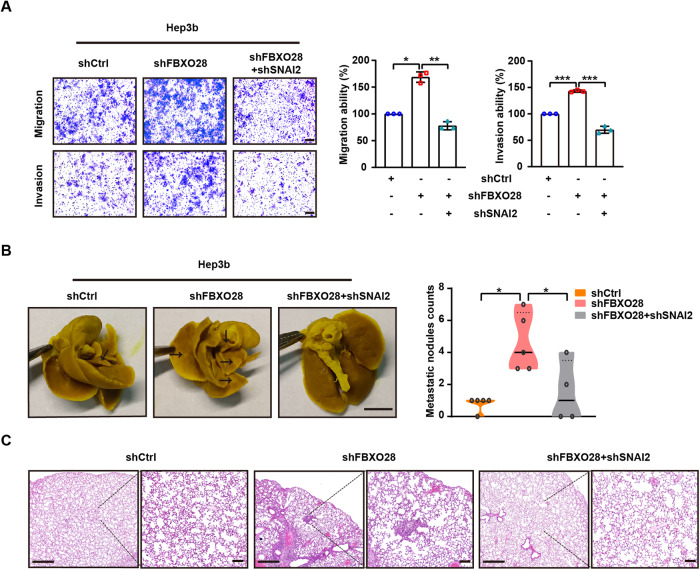
SNAI2 depletion inhibits the enhanced migration, invasion and metastasis capability by FBXO28 knockdown. **A** Hep3b cells stably expressing shCtrl, shFBXO28 alone or in combination with shSNAI2 were subjected to transwell assays. Scale bar: 10 μm. Data were presented as mean ± SD (*n* = 3). *P < 0.05, **P < 0.01 and ***P < 0.001 (one-way ANOVA). **B** Hep3b cells (5 × 10^6^) stably expressing shCtrl, shFBXO28 alone or in combination with shSNAI2 were injected into NOD/SCID mice via tail vein, and the number of lung metastatic nodules was counted after mice were sacrificed at 8 weeks after injection (*n* = 4–5). Scale bar: 4 mm, *P < 0.05 (one-way ANOVA). **C** Hematoxylin and eosin staining of the indicated lung metastatic nodules. The scale bars in left and right panel represent 500 μm and 100 μm, respectively.

To further determine whether FBXO28-mediated SNAI2 degradation is critical for the inhibitory functions of FBXO28 in HCC migration, invasion and metastasis, we compared the biological effects of Hep3b cells stably expressing shFBXO28 alone or in combination with shSNAI2 by lentiviral infection. As seen in Fig. 6A and Supplementary Fig. S6F, the enhanced migration and invasion capability by FBXO28 knockdown was abrogated by depletion of SNAI2. Further in vivo experiment proved SNAI2 knockdown greatly inhibited the increased metastatic pulmonary nodules in FBXO28-depleted Hep3b cells in mice model (Fig. 6B, C). This confirms that the biological function of FBXO28 in the regulation of invasion and metastasis relies on the presence of SNAI2. Collectively, our data suggest PKA-dependent phosphorylation is required for FBXO28-mediated SNAI2 degradation.

### FBXO28 and PKAα protein levels negatively correlate with SNAI2 levels, and low *PRKACA* expression is associated with poor prognosis of liver cancer patients

**Fig. 7:**
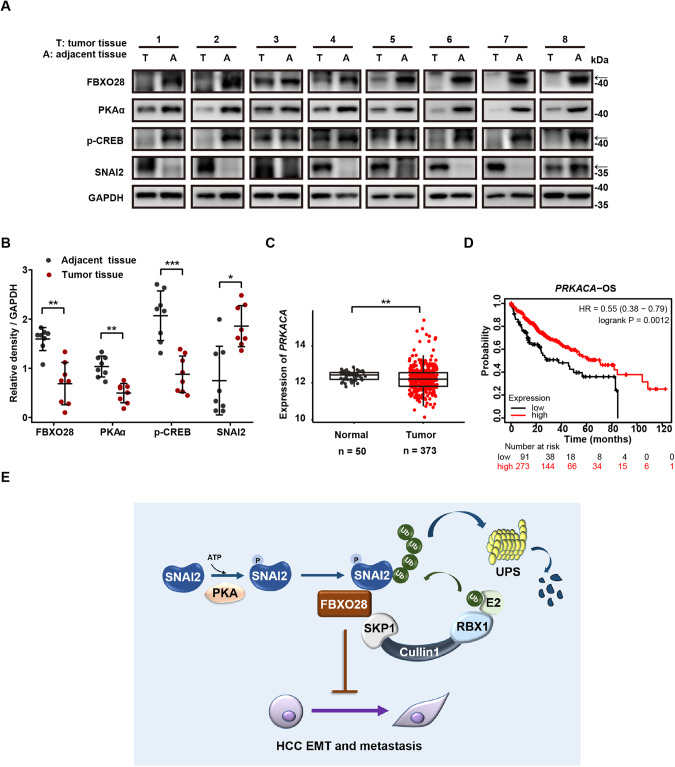
Levels of FBXO28, PKAα, p-CREB and SNAI2 in HCC specimens, and clinical significance of *PRKACA*. **A** Protein levels of FBXO28, PKAα, p-CREB and SNAI2 in paired HCC specimens by IB analyses. Arrows indicate target band. **B** Quantification data of (**A**) were expressed as mean ± SD (*n* = 8). *P < 0.05, **P < 0.01 and ***P < 0.001 (paired *T*-test). **C** Expression of *PRKACA* mRNA levels in HCC and normal cohorts in TCGA dataset. **P < 0.01 (independent *T*-test). **D** Correlation of *PRKACA* expression and overall survival (OS) via Kaplan–Meier survival analysis of liver cancer patients. **E** Working model for the regulatory function of FBXO28 during HCC EMT process by degrading SNAI2 via UPS.

In light of above findings, we lastly assessed and compared the protein levels of FBXO28, SNAI2, PKAα and p-CREB in eight paired HCC specimens obtained from HCC patients. As shown in Fig. 7A, B, FBXO28 protein levels were positively associated with both PKAα and p-CREB levels, all of which were significantly higher in adjacent tissue than that in tumor counterparts. Conversely, SNAI2 levels were much lower in adjacent tissue relative to tumor tissue, showing its negative correlation with FBXO28 and PKAα expression and/or activity. Interestingly, similar as the gene expression pattern of *FBXO28*, a significant downregulation of *PRKACA* was observed in TCGA HCC cohorts as compared to normal counterparts (Fig. 7C); patients with low expression of *PRKACA* is associated with poor OS (Fig. 7D). Collectively, these data support a cooperative function for PKA in regulating FBXO28-mediated SNAI2 degradation.

## Discussion

In the present work, we provide the first evidence to discover FBXO28 as a novel regulator of EMT in HCC. Identified from a transwell assay, FBXO28 has been proved to exert a significant role in suppressing migration, invasion and metastasis in HCC model in vitro and in vivo. FBXO28 also suppresses EMT traits in HCC by positively and negatively regulating epithelial and mesenchymal markers, respectively. Transcriptome analysis reveals an upregulated *FBXO28* expression in a panel of human epithelial cancer cell lines compared to mesenchymal counterparts. Importantly, we establish FBXO28 as a bona fide E3 ubiquitin ligase for SNAI2. By binding to PKA-phosphorylated SNAI2, FBXO28 targets the substrate for UPS-mediated degradation (Fig. 7E).

The E3 ubiquitin ligase of FBXO28 has been found to mediate K48- and K63-linked polyubiquitination, rendering target proteins for proteolytic degradation and stabilization, respectively [12, 13]. So far, several substrates for FBXO28 have been identified, including MYC, HIF-1α, Alcat1 and indirectly, ERK5, under physiological and/or pathological contexts [12, 13, 29, 30]. Meanwhile, self-degradation of FBXO28 is observed [31]. Interestingly, FBXO28 is found to exert either oncogenic or tumor suppressing functions by targeting MYC, SMARCC2 or HIF-1α, under different cellular contexts, complicating its role in tumorigenesis and metastasis [12, 13, 32]. Here, we identify SNAI2 as a novel substrate of FBXO28 based on the following lines of evidence: (1) FBXO28 interacts with SNAI2 under endogenous and exogenous conditions; (2) cellular levels and protein half-life of SNAI2 are increased or decreased by FBXO28 depletion or FBXO28 overexpression, respectively, without affecting its transcriptional expression; (3) FBXO28-SNAI2 binding results in K48-linked SNAI2 polyubiquitination, which is abrogated by FBXO28ΔF mutant, although the mutant fails to rescue SNAI2 by a separately transcriptional mechanism yet to be identified; (4) given that PKA inhibitors abrogate the FBXO28-SNAI2 binding and FBXO28-mediated SNAI2 degradation, our data suggested that PKA could be a kinase that phosphorylates putative S158, S247 and S254 residues at the SNAI2’s binding motif and facilitate its binding to and subsequently degradation by FBXO28. Accordingly, SNAI2 depletion greatly inhibited the increased cell migration, invasion and metastasis capability by FBXO28 knockdown. Therefore, our work reveals SNAI2 as an unidentified substrate of the SCF^FBXO28^ E3 ubiquitin ligase.

As one of main regulators during EMT and metastasis, SNAI2 is often upregulated in various cancer types; high SNAI2 expression normally predicts poor prognosis in cancer patients, including HCC [5, 33, 34]. Until now, a panel of E3 ubiquitin ligases, including β-TrCP1 (also known as FBXW1), FBXL14, CHIP, MDM2 and ASB13, have been identified for controlling SNAI2 degradation in GSK3β-dependent and -independent ways [23, 35–38]. However, none of these studies have revealed the regulatory machinery of homeostatic SNAI2 levels under EMT contexts in HCC. Here, we have identified FBXO28 as a novel E3 ubiquitin ligase to regulate SNAI2 protein stability. Importantly, unlike other E3 ubiquitin ligases that mainly locate in the cytosol (such as β-TrCP1 and FBXL14), we discovered that FBXO28 is primarily present in the nucleus. Considering FBXO28 and SNAI2 co-localize inside the nucleus, it is reasonable to speculate that endogenous SNAI2 may be mainly dependent on FBXO28 for degradation. Whether and how these E3 ubiquitin ligases under different subcellular compartment might coordinate to control the overall cellular SNAI2 abundance remain intriguing. Another interesting finding in this work is that FBXO28 binds to and ubiquitylates SNAI2 at its C-terminal domain, which is different from the previous notion that the N-terminal part of SNAI2 confers its degradation [33, 39]. This suggests an unique feature of FBXO28 in regulating SNAI2 homeostasis. Given that the C-terminal domain is well conserved among the Snail family members, we also detected an interaction between FBXO28 and SNAI1 (data not shown). Notably, since the C-terminal zinc finger domain in SNAI2 facilitates its binding to E-box consensus motifs of target genes such as *CDH1* [33], it is also possible that the FBXO28-SNAI2 binding might disturb the SNAI2’s activity as a transcriptional repressor.

We noticed FBXO28 exerted a significant growth inhibitory effect in HCC cells in vitro whereas only a marginal function in HCC xenograft model. This discrepancy might possibly result from a complicated tumor microenvironment affecting its tumor-suppressing function in vivo. These results indicate that FBXO28 may primarily function as a suppressor of HCC metastasis instead of tumorigenesis. Supportively, clinical data suggest a more significant role for *FBXO28* expression in predicting RFS instead of OS in HCC cohorts. Interestingly, the disparities between metastatic and tumorigenic function have also been demonstrated for another F-box protein FBXO22, which functions as an E3 ligase controlling SNAI1 degradation [40].

Previous studies have identified several kinases that can phosphorylate SNAI2 protein, including GSK3β, CDK2 and PAK1 as negative or positive players [23, 26, 27, 36]. Here, we have found PKA-dependent phosphorylation serves an unidentified and pivotal role in regulating FBXO28 mediated SNAI2 decay. Inhibiting PKA activity or mutating PKA putative phosphorylation sites on SNAI2 disrupts the interaction of SNAI2 with FBXO28 and stabilizes the substrate, thereby promoting EMT in HCC. Our findings are complementary with previously established anti-metastatic functional function of PKA during EMT process [41–43]. Importantly, we found a positive correlation among FBXO28, PKA and its substrate p-CREB protein levels whereas a negative correlation of these proteins with SNAI2 in HCC specimens. Moreover, clinical data analysis reveals that *FBXO28* and *PRKACA* gene are similarly downregulated in HCC patients; low expression of either one is associated with poor prognosis. These data support PKA’s function as a positive player of FBXO28. More work is needed to clarify the mechanism to address how *FBXO28* and *PRKACA* genes are downregulated in HCC.

Collectively, our findings establish the unrevealed function of FBXO28 as a critical inhibitor of invasion and metastasis in HCC by promoting SNAI2 degradation. FBXO28 may serve as a novel prognostic marker and/or therapeutic target for aggressive HCC.

## Materials and methods

### Cell lines

Huh7, HCCLM3, Hep3b, NCI-H460 and Saos-2 cell lines were purchased from the national infrastructure of cell line resources in China. HEK293T and MDA-MB-231 cell lines were obtained from ATCC. All cell lines were maintained under standard conditions and authenticated by STR profiling on a regular basis every half-year.

### Antibodies and reagents

Anti-FBXO28 (#ab154068) and anti-p-CREB (S133) (#ab32096) antibodies were purchased from Abcam. Anti-GAPDH (#60004-1-Ig) and anti-β-actin (#66009-1-Ig) antibodies were obtained from Proteintech. Anti-IgG (#sc-2027), anti-SNAI2 (#sc-166476) and anti-PKAα (#sc-28315) antibodies were from Santa Cruz Biotechnology. Anti-SNAI1 (#3879), anti-SNAI2 (#9585), anti-Vimentin (#5741), anti-E-cadherin (#3195), anti-Ubiquitin (#3936), anti-CREB (48H2) (#9197), anti-HA (#3724), anti-DYKDDDDK (Flag) (#14793), anti-Myc (#2278), anti-CULLIN1 (#4995), anti-SKP1 (#12248), Rabbit Anti-Mouse IgG (Light-Chain Specific) (#58802), Mouse Anti-Rabbit IgG (Light-Chain Specific) (#93702), Mouse Anti-rabbit IgG (Conformation Specific) (#5127) antibodies were purchased from Cell Signaling Technology. The HRP-labeled Goat Anti-Rabbit IgG (H + L) (#ZB-2301), HRP-labeled Goat Anti-Mouse IgG (H + L) (#ZB-5305), Alexa Fluor^®^ 488-labeled Goat Anti-Mouse IgG (H + L) (#ZF-0512), Alexa Fluor^®^ 594-labeled Goat Anti-Rabbit IgG (H + L) (#ZF-0516) and DAPI (#ZLI-9557) were obtained from ZSGB-BIO (China). The anti-Myc (#B26302), anti-HA (#B26202) and anti-Flag (#B26102) magnetic beads were from Bimake (China). Cycloheximide (#S7418), MG132 (#S2619), H89 (#S1582), CHIR-99021 (#S1263), CID755673 (#S7188), PF-3758309 (#S7094) and Dinaciclib (#S2768) were purchased from Selleckchem (China). KT5720 (#HY-N6789) and puromycin (#HY-B1743A) were obtained from MedChemExpress.

### Plasmids and siRNAs

pcDNA3.1-SNAI2-Myc (#31698) was obtained from Addgene. pCMV6-FBXO28-Myc-Flag (#RC206490), pLenti-C-mGFP-P2A (#PS100093), pLenti-FBXO28-mGFP-P2A (#RC206490L4), pRS-shSNAI2 (#TR309225B) and pRS-shRNA control (#TR30012) plasmids were acquired from Origene Technologies. pcDNA4.0-SNAI2-HA and pLKO.1-shFBXO28 plasmids, were constructed in our lab. FBXO28 and SNAI2 deletion or point mutants were obtained from Sangon Biotech Co. Ltd, China. The His-Ub K48R (#P45617) and His-Ub K63R (#45929) plasmids were obtained from MiaoLing Biology (China). CRISPR/Cas9 based FBXO28 knockout plasmid (YKO-RP003-hFBXO28) was obtained from the UBIGENE Biosciences in China. The pooled siRNAs oligos targeting for a panel of F-box proteins were synthesized by Beijing Cornerstone Life Science and Technology Co. Ltd, China. The target sequences of siRNAs, shFBXO28 and guide RNA targeting *FBXO28* exon 1 were listed in Supplementary Table 1.

### Cell transfection and infection

The siRNAs or plasmids were transfected into cells using lipofectamine 2000 (Thermo, #11668019) or DNA transfection reagent (Neofect, #TF20121201), respectivel. For cell infection, the pLKO.1-shFBXO28 or pLKO.1 control vector; pLenti-FBXO28-mGFP-P2A or pLenti-mGFP-P2A; pRS-shSNAI2 or pRS-shRNA control was co-transfected into HEK293T cells with lentivirus package plasmids (psPAX2 and pMD2.0 G) to produce viruses for stable knockdown or expression of FBXO28, or knockdown of SNAI2, respectively. The supernatant containing viruses were collected at 72 h after transfection for infection. Stably infected cell lines were selected and maintained at 5 μg/mL puromycin.

To generate a CRISPR/Cas9-mediated FBXO28 knockout cell line (FBXO28^KO^), Huh7 cells were transfected with YKO-RP003-hFBXO28 using lipofectamine 2000. Target cells were screened at 72 h after transfection with 5 μg/mL puromycin. Limited dilution was performed to isolate monoclonal knockout cells. The deletion efficiency of FBXO28 was confirmed by PCR sequencing and immunoblotting.

### Migration and invasion assay

Migration capability was assessed by wound healing and transwell assay [44]. In wound-healing assay, Huh7 cells transfected with the indicated plasmid for 48 h were seeded into 96-well plates (2 × 10^4^ cells/well) in triplicate, and confluent cell monolayers were wounded by automatically scratching, and subsequently cultured in serum-free medium for the indicated time. The images were captured using BioTek Lionheart FX, Agilent, and wound area analysis was processed by Gen5 Image+ 3.12. Cell number under the same conditions without scratching was counted.

Transwell and matrigel invasion chambers (#3422 and #354480, Corning) were used for measurement of cell migrative and invasive ability, respectively. Huh7 (5 × 10^4^/well for migration and 1.5 × 10^5^/well for invasion) or Hep3b (2 × 10^5^/well for migration and 2.5 × 10^5^/well for invasion) cells were resuspended in serum-free culture medium and added to upper chambers, while culture medium containing 20% FBS was added to lower chambers. After incubation for the indicated time, the chambers were fixed with 4% paraformaldehyde (#P1110, Solarbio, China) for 30 mins and stained with 0.1% (w/v) crystal violet (#G1064, Solarbio) for 30 mins. Data were quantified by dissolving the stain in acetic acid and measuring absorbance at 570 nm using a microplate reader (BIO-RAD). Cells were treated under the same serum-free conditions as that in the migration assay and plated into 96-well plates for ATPlite luminescence measurement (#6016941, PerkinElmer) following the kit instructions to determine cell viability changes during the migrative process. Cell number was counted under the same conditions as in the migration assay to measure proliferation changes.

### Colony formation

Cells stably expressing Flag- and Myc-tagged FBXO28 (FBXO28-Flag-Myc, abbreviated as FBXO28-Flag throughout the work) or shFBXO28 were seeded at 2 × 10^3^ cells/well onto 6-well plates and cultured in full-serum medium. After 2 weeks, the colonies were stained with 0.1% (w/v) crystal violet (#G1064, Solarbio) and counted.

### Animal study

For xenograft model, 6–8-week-old female BALB/c nude mice (SPF Biotechnology Co. Ltd, China) were randomly divided into two groups (with six in each group). HCCLM3 (5 × 10^6^) or Hep3b (8 × 10^6^) cells were inoculated into the right axilla of the mice. The body weight of the mice, length (a) and width (b) of tumors were measured every 2 days during the experiment. All mice were sacrificed at the 18th (for HCCLM3) or 30th (for Hep3b) day post inoculation, and the tumor tissues were collected, photographed, and weighted. For in vivo tumor metastasis model, 5-week-old female NOD/SCID mice (SPF Biotechnology Co. Ltd, China) were randomly divided equally into different groups (with five in each group), and 200 μl of cell suspension containing 2 × 10^6^ HCCLLM3 or 5 × 10^6^ Hep3b cells was injected into mice via tail vein. After 10 (HCCLLM3 cells) or 8 (Hep3b cells) weeks, mice were sacrificed. The lung and heart tissue were removed and fixed in Bouin’s solution (#HT10132, Sigma-Aldrich). Metastatic nodules in lungs were counted prior to hematoxylin and eosin staining. All animal experiments were approved by the ethics committee of our institute, and conducted in accordance with the regulations and operational procedures of experimental animal management. During these procedures, measurement and analyses were performed in a double-blinded manner.

### Immunoblotting (IB), immunoprecipitation (IP) and immunofluorescence (IF)

IB analyses were performed using indicated antibodies as described before [45]. For protein half-life analysis, cells were treated with 10 μg/mL CHX for the indicated time prior to IB analysis. For IP analysis, cells were transfected with indicated plasmids, treated with MG132 (20 μM) for 6 h prior to harvest and lysed with Triton X-100 buffer (150 mM NaCl, 50 mM Tris, and 1% Triton X-100, pH 7.5). After incubation with 20 μl anti-Myc/anti-Flag/anti-HA magnetic beads or 20 μl Protein A/G magnetic beads containing 4 μl indicated antibodies overnight at 4 °C, the immunocomplexes were washed five times with lysis buffer and analyzed by IB. For IF analysis, cells were seeded on coverslips and fixed with 4% paraformaldehyde. The proteins were stained with respective antibodies and cell nuclei were stained with DAPI.

### Ubiquitination assay

The in vivo ubiquitination assay was performed as described before [46]. HEK293T cells were co-transfected with the indicated plasmids for 72 h. After treatment with MG132 (20 μM) for 6 h, the cells were collected and lysed with RIPA buffer containing 1% SDS supplemented with protease inhibitors (Roche, #04693116001). Cell lysates were briefly sonicated, boiled at 95 °C for 10 min, and then centrifuged at 12,000 *rpm* for 15 min. The supernatants were then diluted 1:9 with RIPA lysis buffer to reduce the SDS concentration to 0.1%. Anti-HA magnetic beads were incubated with diluted lysates overnight at 4 °C. Immunocomplexes were washed five times with RIPA lysis buffer and analyzed by IB.

For the Ni-nitrilotriacetic acid (NTA) capture assay, 20% of the cells were harvested and lysed with RIPA lysis buffer. After sonicating and centrifuging, the lysates were added with 5 × loading buffer and boiled for 10 min at 100 °C and stored at −80 °C as input samples. The remaining cells were lysed in 1 ml highly denaturing buffer A1 (6 M guanidium-HCl, 10 mM Tris-HCl, 100 mM Na_2_HPO_4_/NaH_2_PO_4_, 5 mM imidazole, pH 8.0). After sonicating and incubating on the ice for 30 min, the lysis solution was added with 50 μl Ni-NTA His-tag Purification Agarose (MCE, #HY-K0210) and incubated at 4 °C for 16 h. The beads-protein complex was washed with buffer A2 (6 M guanidium-HCl, 10 mM Tris-HCl, 100 mM Na_2_HPO_4_/NaH_2_PO_4_, 10 mM β-mercaptoethanol, pH 8.0), buffer B (10 M Urea, 10 mM Tris-HCl, 100 mM Na_2_HPO_4_/NaH_2_PO_4_, 10 mM β-mercaptoethanol, pH 8.0), buffer C1 (8 M Urea, 10 mM Tris-HCl, 100 mM Na_2_HPO_4_/NaH_2_PO_4_, 10 mM β-mercaptoethanol, 0.2% Triton X-100, pH 6.3), and buffer C2 (8 M Urea, 10 mM Tris-HCl, 100 mM Na_2_HPO_4_/NaH_2_PO_4_, 10 mM β-mercaptoethanol, 0.1% Triton X-100, pH 6.3), respectively. After washing, the ubiquitinated proteins were eluted with 50 μl 1 × elution buffer (20 mM pH 6.8 Tris-HCl, 10% Glycerine, 0.8% SDS, 0.1% Bromophenol blue, 720 mM β-mercaptoethanol, 300 mM imidazole) and boiled at 100 °C for 5 min. The complex was centrifuged at 12000 *rpm* for 2 min and supernatants were collected prior to IB analysis.

For in vitro ubiquitylation assay, HEK293T cells were transfected with pCMV6-FBXO28-Myc-Flag plasmids for 48 h prior to MG132 treatment for 6 h, and lysed with Triton X-100 buffer as for IP. The cell lysates were incubated with 20 μl anti-Myc magnetic beads overnight at 4 °C. After washing with lysis buffer, the SCF^FBXO28^ immunocomplexes were added into the incubation reaction system comprised of E1 ubiquitin-activating enzyme, E2 ubiquitin-conjugation enzyme, SNAI2 protein (#TP760008, Origene), ATP and Ubiquitin according to instructions in the Ubiquitination Kit (#BML-UW9920, Enzolife).

### Quantitative RT-PCR (qRT-PCR) analysis

qRT-PCR was conducted as described previously [47], with primers sequences in Supplementary Table 2.

### Human tissue analysis

Human HCC tissue array (No. HLivH060CD03) was purchased from Shanghai Outdo Biotech (China) and subjected to immunohistochemistry (IHC) analysis with antibody against FBXO28 (#24282-1-AP, Proteintech) at 1:50 dilution. The staining intensity was classified into five groups with increasing staining intensity from marginal (10 points) to the strongest (30 points). The paired HCC specimens were collected from the Qilu Hospital of Shandong University, China, with written informed consents obtained from all patients based on the Declaration of Helsinki. The study was approved by Research Ethics Committee of Qilu Hospital and our Institute’s Ethics Committee before subjected to IB analysis.

### Statistical analysis

Data are presented as mean ± SD. Statistical analysis was performed using independent *T*-test (between two groups) or one-way ANOVA analysis (within multiple groups) for data comparison using SPSS (version Statistic 25). Representative IB data from at least three independent experiments are shown. Differences are considered significant at three levels: *P < 0.05, **P < 0.01 and ***P < 0.001.

### Supplementary information

Supplementary Data

Video1-Huh7-Vector-wound healing

Video2-Huh7-FBXO28-Flag-wound healing

## Data Availability

The data in this study are available upon request from the corresponding author.

## References

[1] Oura K, Morishita A, Tani J, Masaki T (2021). Tumor immune microenvironment and immunosuppressive therapy in hepatocellular carcinoma: a review. Int J Mol Sci.

[2] Aiello NM, Kang Y (2019). Context-dependent EMT programs in cancer metastasis. J Exp Med.

[3] Brabletz S, Schuhwerk H, Brabletz T, Stemmler MP (2021). Dynamic EMT: a multi-tool for tumor progression. EMBO J..

[4] Shanmugam MK, Chong PP, Looi CY (2019). The E-Cadherin and N-cadherin switch in epithelial-to-mesenchymal transition: signaling, therapeutic implications, and challenges. Cells..

[5] Yastrebova MA, Khamidullina AI, Tatarskiy VV, Scherbakov AM (2021). Snail-family proteins: role in carcinogenesis and prospects for antitumor therapy. Acta Naturae.

[6] Rodríguez-Alonso A, Casas-Pais A, Roca-Lema D, Graña B, Romay G, Figueroa A (2020). Regulation of Epithelial-Mesenchymal plasticity by the E3 ubiquitin-ligases in cancer. Cancers (Basel).

[7] Díaz VM, de Herreros AG (2016). F-box proteins: Keeping the epithelial-to-mesenchymal transition (EMT) in check. Semin Cancer Biol.

[8] Inoue Y, Itoh Y, Sato K, Kawasaki F, Sumita C, Tanaka T (2016). Regulation of epithelial-mesenchymal transition by E3 ubiquitin ligases and deubiquitinase in cancer. Curr Cancer Drug Targets.

[9] Hanus J, Kalinowska-Herok M, Widlak P (2010). Identification of novel putative regulators of the major apoptotic nuclease DNA Fragmentation Factor. Acta Biochim Pol.

[10] Au PY, Argiropoulos B, Parboosingh JS, Micheil Innes A (2014). Refinement of the critical region of 1q41q42 microdeletion syndrome identifies FBXO28 as a candidate causative gene for intellectual disability and seizures. Am J Med Genet A..

[11] Kratz AS, Richter KT, Schlosser YT, Schmitt M, Shumilov A, Delecluse HJ (2016). Fbxo28 promotes mitotic progression and regulates topoisomerase IIα-dependent DNA decatenation. Cell Cycle..

[12] Cepeda D, Ng HF, Sharifi HR, Mahmoudi S, Cerrato VS, Fredlund E (2013). CDK-mediated activation of the SCF^FBXO28^ ubiquitin ligase promotes MYC-driven transcription and tumourigenesis and predicts poor survival in breast cancer. EMBO Mol Med.

[13] Phillips E, Balss J, Bethke F, Pusch S, Christen S, Hielscher T (2022). PFKFB4 interacts with FBXO28 to promote HIF-1α signaling in glioblastoma. Oncogenesis..

[14] Liu Y, Pan B, Qu W, Cao Y, Li J, Zhao H (2021). Systematic analysis of the expression and prognosis relevance of FBXO family reveals the significance of FBXO1 in human breast cancer. Cancer Cell Int.

[15] Zhang Y, Liu Q, Cui M, Wang M, Hua S, Gao J (2022). Comprehensive analysis of expression, prognostic value, and immune infiltration for ubiquitination-related fbxos in pancreatic ductal adenocarcinoma. Front Immunol.

[16] Fagerholm R, Khan S, Schmidt MK, García-Closas M, Heikkilä P, Saarela J (2017). TP53-based interaction analysis identifies cis-eQTL variants for TP53BP2, FBXO28, and FAM53A that associate with survival and treatment outcome in breast cancer. Oncotarget..

[17] Sahu SK, Tiwari N, Pataskar A, Zhuang Y, Borisova M, Diken M (2017). FBXO32 promotes microenvironment underlying epithelial-mesenchymal transition via CtBP1 during tumour metastasis and brain development. Nat Commun.

[18] Liu H, Radisky DC, Yang D, Xu R, Radisky ES, Bissell MJ (2012). MYC suppresses cancer metastasis by direct transcriptional silencing of αv and β3 integrin subunits. Nat Cell Biol.

[19] Rieser E, Cordier SM, Walczak H (2013). Linear ubiquitination: a newly discovered regulator of cell signalling. Trends Biochem Sci.

[20] Erpapazoglou Z, Walker O, Haguenauer-Tsapis R (2014). Versatile roles of k63-linked ubiquitin chains in trafficking. Cells..

[21] Zou S, Ma C, Yang F, Xu X, Jia J, Liu Z (2018). FBXO31 suppresses gastric cancer emt by targeting snail1 for proteasomal degradation. Mol Cancer Res.

[22] Xu M, Zhu C, Zhao X, Chen C, Zhang H, Yuan H (2015). Atypical ubiquitin E3 ligase complex Skp1-Pam-Fbxo45 controls the core epithelial-to-mesenchymal transition-inducing transcription factors. Oncotarget..

[23] Wu ZQ, Li XY, Hu CY, Ford M, Kleer CG, Weiss SJ (2012). Canonical Wnt signaling regulates Slug activity and links epithelial-mesenchymal transition with epigenetic Breast Cancer 1, Early Onset (BRCA1) repression. Proc Natl Acad Sci USA.

[24] Zheng H, Shen M, Zha YL, Li W, Wei Y, Blanco MA (2014). PKD1 phosphorylation-dependent degradation of SNAIL by SCF-FBXO11 regulates epithelial-mesenchymal transition and metastasis. Cancer Cell.

[25] MacPherson MR, Molina P, Souchelnytskyi S, Wernstedt C, Martin-Pérez J, Portillo F (2010). Phosphorylation of serine 11 and serine 92 as new positive regulators of human Snail1 function: potential involvement of casein kinase-2 and the cAMP-activated kinase protein kinase A. Mol Biol Cell.

[26] Wang WL, Huang HC, Kao SH, Hsu YC, Wang YT, Li KC (2015). Slug is temporally regulated by cyclin E in cell cycle and controls genome stability. Oncogene..

[27] Thaper D, Vahid S, Nip KM, Moskalev I, Shan X, Frees S (2017). Targeting Lyn regulates Snail family shuttling and inhibits metastasis. Oncogene..

[28] Xu J, Zhou W, Yang F, Chen G, Li H, Zhao Y (2017). The β-TrCP-FBXW2-SKP2 axis regulates lung cancer cell growth with FBXW2 acting as a tumour suppressor. Nat Commun.

[29] Zou C, Synan MJ, Li J, Xiong S, Manni ML, Liu Y (2016). LPS impairs oxygen utilization in epithelia by triggering degradation of the mitochondrial enzyme Alcat1. J Cell Sci.

[30] Yu T, Wang L, Zhao C, Qian B, Yao C, He F (2019). Sublytic C5b-9 induces proliferation of glomerular mesangial cells via ERK5/MZF1/RGC-32 axis activated by FBXO28-TRAF6 complex. J Cell Mol Med.

[31] Cai L, Liu L, Li L, Jia L (2020). SCF^FBXO28^-mediated self-ubiquitination of FBXO28 promotes its degradation. Cell Signal.

[32] Liu S, Liu P, Zhu C, Yang R, He Z, Li Y (2023). FBXO28 promotes proliferation, invasion, and metastasis of pancreatic cancer cells through regulation of SMARCC2 ubiquitination. Aging (Albany NY).

[33] Zhou W, Gross KM, Kuperwasser C (2019). Molecular regulation of Snai2 in development and disease. J Cell Sci.

[34] Meng J, Ai X, Lei Y, Zhong W, Qian B, Qiao K (2019). USP5 promotes epithelial-mesenchymal transition by stabilizing SLUG in hepatocellular carcinoma. Theranostics..

[35] Lander R, Nordin K, LaBonne C (2011). The F-box protein Ppa is a common regulator of core EMT factors Twist, Snail, Slug, and Sip1. J Cell Biol.

[36] Kao SH, Wang WL, Chen CY, Chang YL, Wu YY, Wang YT (2014). GSK3β controls epithelial-mesenchymal transition and tumor metastasis by CHIP-mediated degradation of Slug. Oncogene..

[37] Wang SP, Wang WL, Chang YL, Wu CT, Chao YC, Kao SH (2009). p53 controls cancer cell invasion by inducing the MDM2-mediated degradation of Slug. Nat Cell Biol.

[38] Fan H, Wang X, Li W, Shen M, Wei Y, Zheng H (2020). ASB13 inhibits breast cancer metastasis through promoting SNAI2 degradation and relieving its transcriptional repression of YAP. Genes Dev.

[39] Vernon AE, LaBonne C (2006). Slug stability is dynamically regulated during neural crest development by the F-box protein Ppa. Development..

[40] Sun R, Xie HY, Qian JX, Huang YN, Yang F, Zhang FL (2018). FBXO22 possesses both protumorigenic and antimetastatic roles in breast cancer progression. Cancer Res.

[41] Pattabiraman DR, Bierie B, Kober KI, Thiru P, Krall JA, Zill C (2016). Activation of PKA leads to mesenchymal-to-epithelial transition and loss of tumor-initiating ability. Science..

[42] Ognjenovic NB, Bagheri M, Mohamed GA, Xu K, Chen Y, Mohamed Saleem MA (2020). Limiting self-renewal of the basal compartment by PKA activation induces differentiation and alters the evolution of mammary tumors. Dev Cell.

[43] Ko FC, Chan LK, Sze KM, Yeung YS, Tse EY, Lu P (2013). PKA-induced dimerization of the RhoGAP DLC1 promotes its inhibition of tumorigenesis and metastasis. Nat Commun.

[44] Lu M, Zhu WW, Wang X, Tang JJ, Zhang KL, Yu GY (2019). ACOT12-Dependent Alteration of Acetyl-CoA Drives Hepatocellular Carcinoma Metastasis by Epigenetic Induction of Epithelial-Mesenchymal Transition. Cell Metab.

[45] Sha MQ, Zhao XL, Li L, Li LH, Li Y, Dong TG (2016). EZH2 mediates lidamycin-induced cellular senescence through regulating p21 expression in human colon cancer cells. Cell Death Dis.

[46] Wang Q, Li H, Tajima K, Verkerke ARP, Taxin ZH, Hou Z (2022). Post-translational control of beige fat biogenesis by PRDM16 stabilization. Nature..

[47] Shen J, Li P, Shao X, Yang Y, Liu X, Feng M (2018). The E3 ligase RING1 targets p53 for degradation and promotes cancer cell proliferation and survival. Cancer Res.

