# Supplementary material for: FBXO28 suppresses liver cancer invasion and metastasis by promoting PKA-dependent SNAI2 degradation
Source: Oncogene. 2023 Aug 18;42(39):2878–91. doi: 10.1038/s41388-023-02809-0 (PMC10516749; doi:10.1038/s41388-023-02809-0)
Supplement: Supplementary file 1 — Supplementary Data [file 41388_2023_2809_MOESM1_ESM.docx]

**FBXO28 suppresses liver cancer invasion and metastasis by promoting PKA-dependent SNAI2 degradation**

Xinran Qiao^1#^, Jingyu Lin^1#^, Jiajia Shen^1^, Yang Chen^1^, Liyun Zheng^1^, Hangjiang Ren^1^, Xiaoli Zhao^1^, Hang Yang^2^, Pengyu Li^3^, Zhen Wang^1^*

1 Institute of Medicinal Biotechnology, Chinese Academy of Medical Sciences and Peking Union Medical College, Beijing, China.

2 The Affiliated Cancer Hospital of Zhengzhou University & Henan Cancer Hospital, Zhengzhou, Henan Province, China.

3 Qilu Hospital of Shan Dong University, Jinan, Shandong Province, China.

# The authors have equal contributions to this work.

* Correspondence to: Zhen Wang, Ph. D, Department of Biochemistry, Institute of Medicinal Biotechnology, Chinese Academy of Medical Sciences and Peking Union Medical College, 1# Tian Tan Xi Li, Beijing 100050, China. E-mail: [wangzhen@imb.pumc.edu.cn](mailto:wangzhen@imb.pumc.edu.cn).

**Supplementary Data**

**
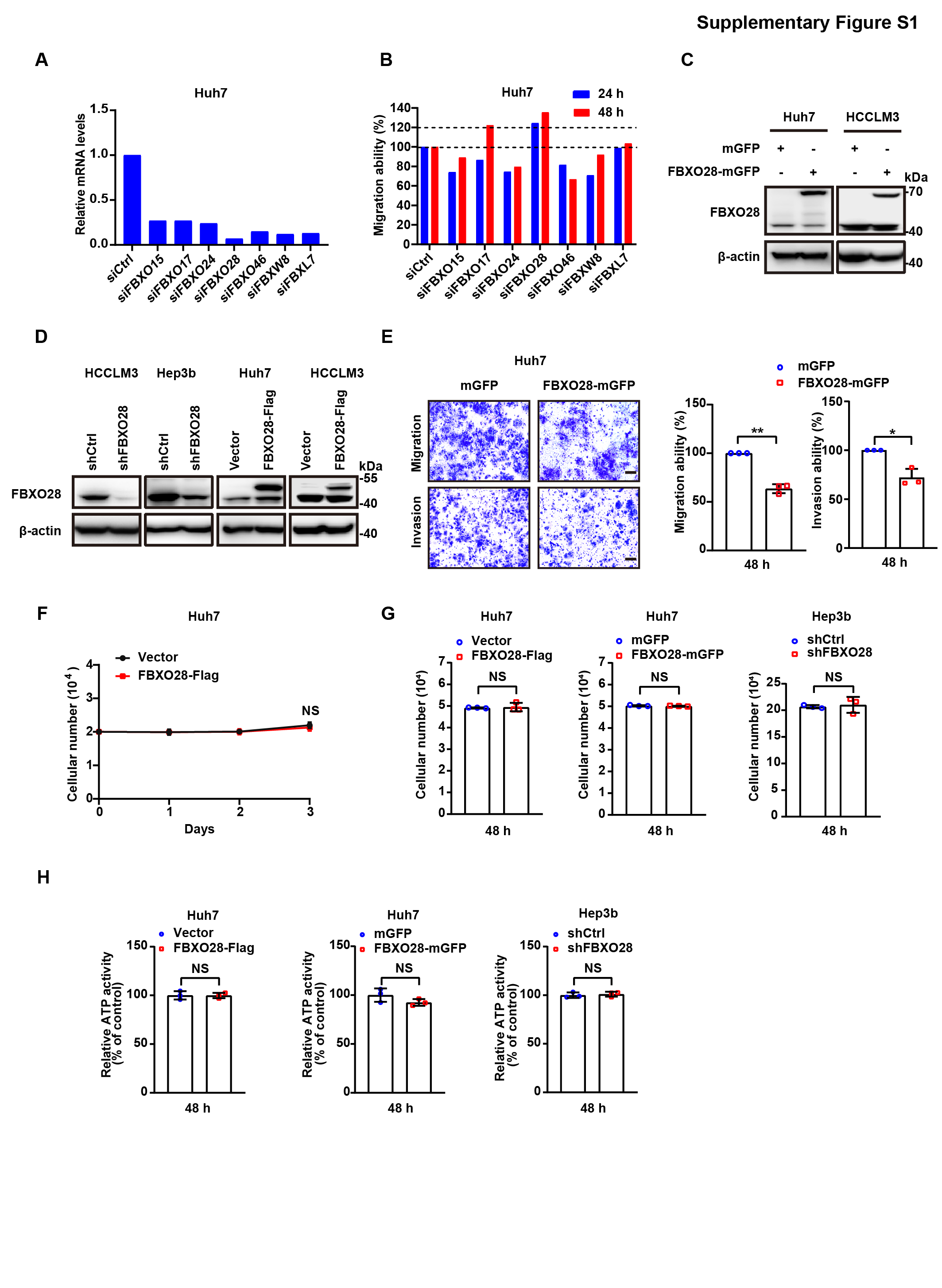
**

**Supplementary Figure S1**. **A.** Knockdown efficiency of a panel of F-box family members. Huh7 cells were transfected with specific siRNAs or siCtrl for 48 hours, followed by RT-PCR analysis. **B.** Huh7 cells were transfected with the specific siRNAs for 48 hours, followed by migration assay for 24 and 48 hours, respectively. **C.** Huh7 and HCCLM3 cells were infected with lentivirus expressing FBXO28-mGFP or mGFP control and stably selected prior to IB analyses. **D.** HCCLM3 and Hep3b cells were infected with lentivirus expressing shFBXO28 or shCtrl and stably selected; or Huh7 and HCCLM3 cells were transfected with pCMV6-FBXO28-Myc-Flag (FBXO28-Flag) or a vector control for 48 hours, followed by IB analyses. **E.** Huh7 cells stably expressing FBXO28-mGFP or mGFP were subjected to migration and invasion assays for the indicated time. Data were shown as mean ± SD (n = 3). ^*^*P* < 0.05, ^**^*P* < 0.01 (independent T test). **F.** Cell number under the same conditions as wound-healing assay (without scratching) was counted. **G.** Cell number was counted under the same conditions as migration assay for 48 hours. **H.** Cells were treated under the same conditions as migration assay for 48 hours and plated into 96-well plates for ATPlite assay. Data in respective vector control group was set as 100%. Data in F-H were shown as mean ± SD from three independent experiments. NS, no significance (independent T test).


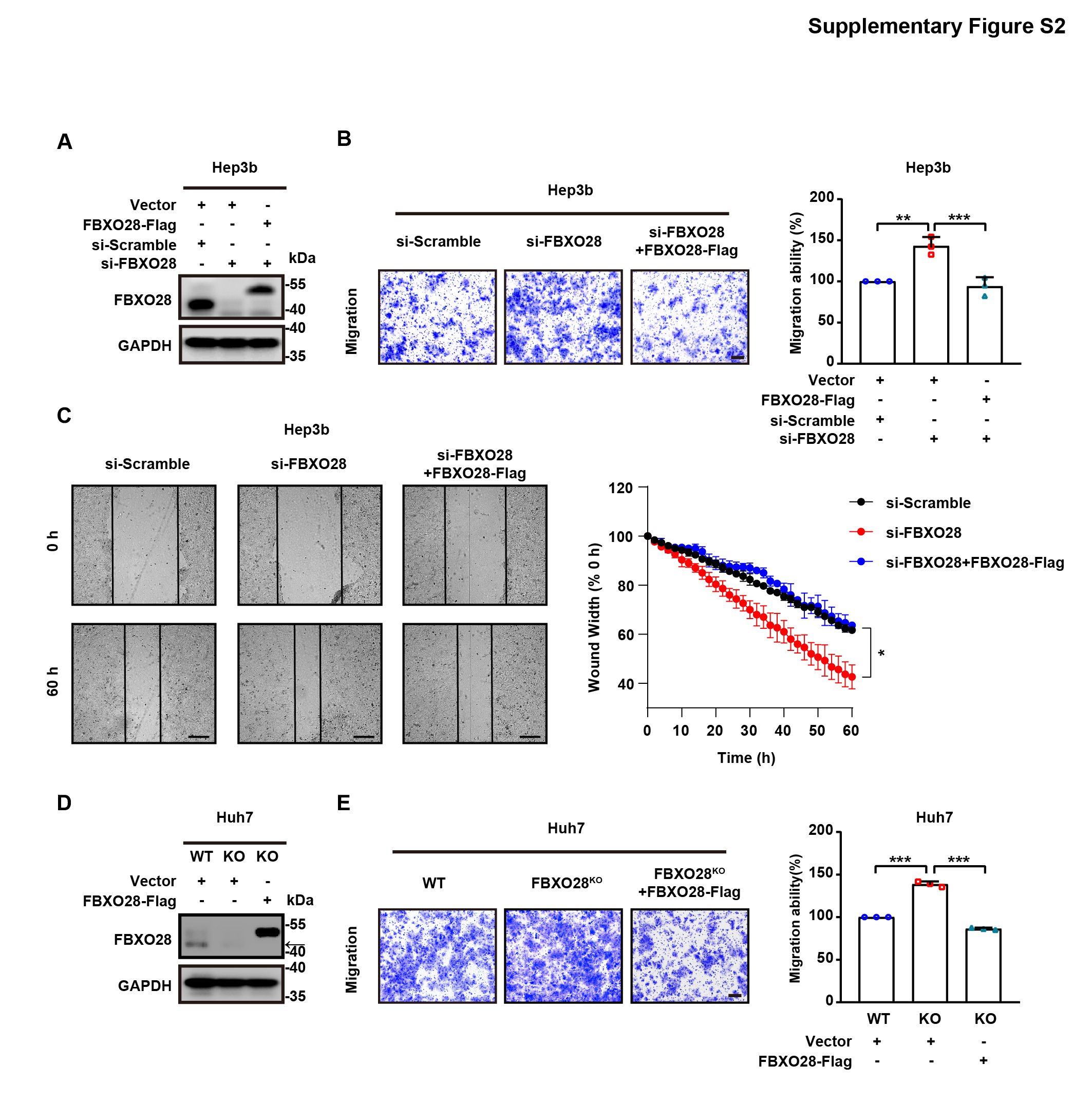


**Supplementary Figure S2. A-C.** Hep3b cells were transfected with si-FBXO28 or si-Scramble for 4 hours, followed by transfection with FBXO28-Flag or a vector control for another 48 hours, and then subjected to IB (A), transwell migration (B) and wound healing (C) assays. Representative images are shown in B after 48 hours (Scale bar: 10 μm) and in C after 60 hours (Scale bar: 0.5 mm), respectively. **D, E.** Huh7 cells depleted of FBXO28 by sgRNA (sg-FBXO28) were re-expressed with FBXO28-Flag or a vector control for 48 hours prior to IB (D) and transwell migration (E) assays. Representative images in E after 48 hours are shown (Scale bar: 10 μm). Quantification data in B, C and E were presented as mean ± SD from three independent experiments. ^*^*P* < 0.05, ^**^*P* < 0.01 and ^***^*P* < 0.001 (one-way ANOVA).

**
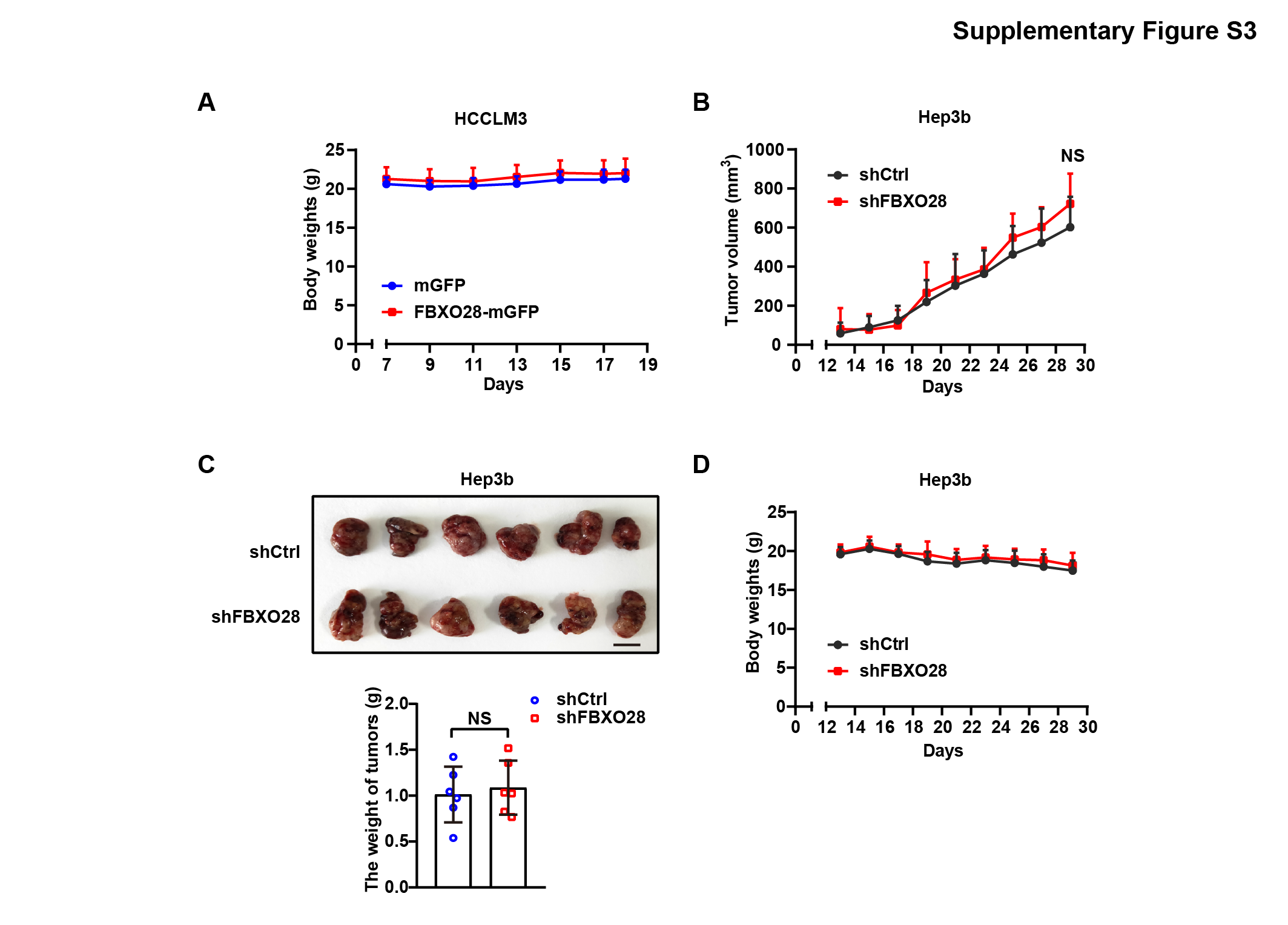
**

**Supplementary Figure S3. A.** The body weight of mice was measured every two days during the indicated time and plotted (n = 6 per group). **B-D.** Hep3b cells stably expressing shFBXO28 or shCtrl were inoculated into BALB/c nude mice. The tumor volume and body weight of mice were measured every two days during the indicated time and plotted in (B) and (D), respectively. Average tumor weight on the day 30^th^ after inoculation were calculated (C). Data were shown as mean ± SD (n = 6). NS, no significance (independent T test).


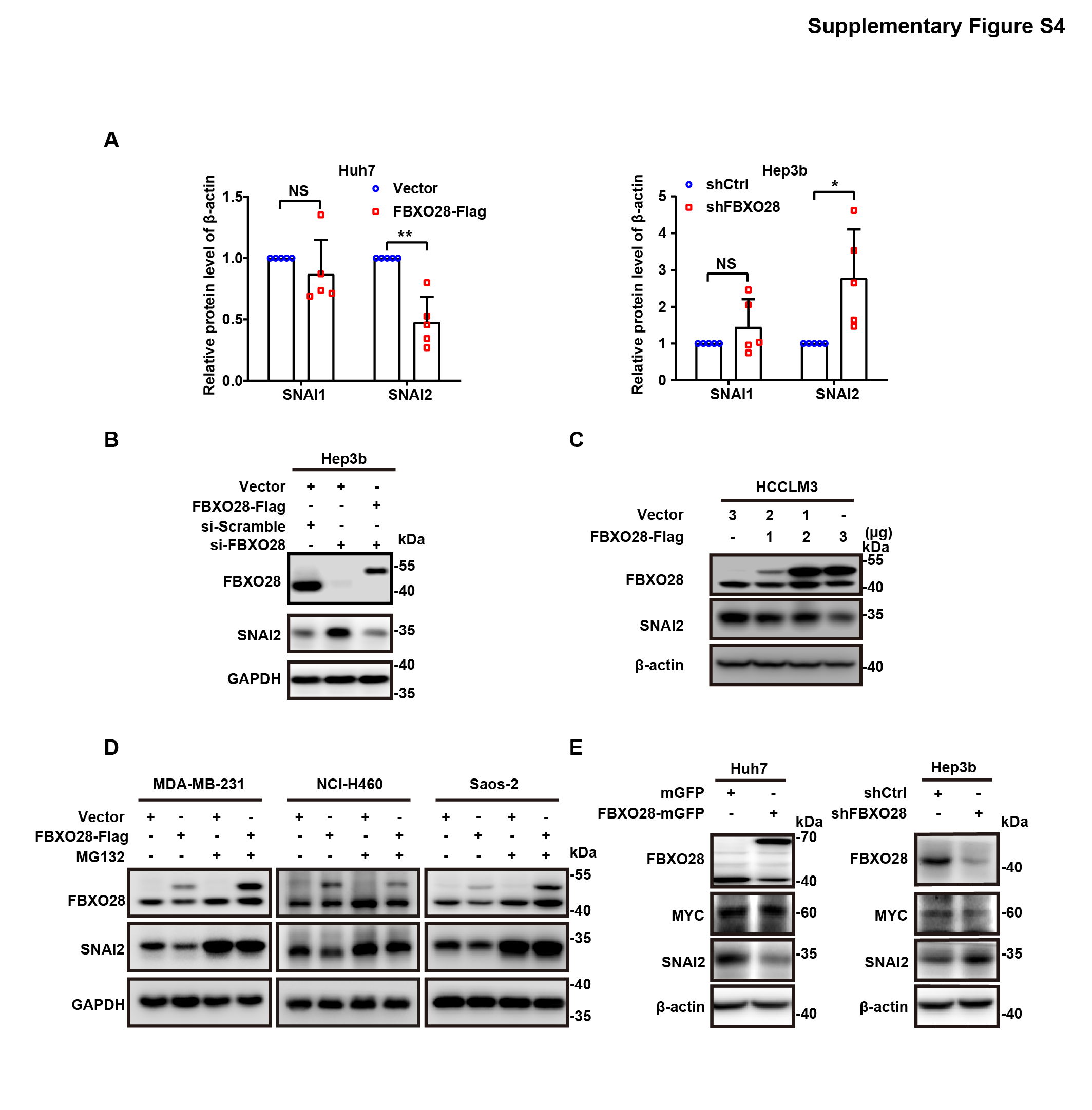
**Supplementary Figure S4**. **A.** Quantification of the protein levels of SNAI1 and SNAI2 in Huh7 cells overexpressing FBXO28-Flag or a control vector for 48 hours or in Hep3b cells stably expressing shFBXO28 or shCtrl for 72 hours. Data were shown as mean ± SD (n = 5). ^*^*P* < 0.05 and ^**^*P* < 0.01 (independent T test). NS: no significance. **B.** Hep3b cells expressing si-Scramble or si-FBXO28 along with FBXO28-Flag or a control vector by transient co-transfection for 48 hours, followed by IB analyses. **C.** HCCLM3 cells were transfected with increasing doses of FBXO28-Flag expressing plasmids for 72 hours, followed by IB analyses. **D.** Cells expressing FBXO28-Flag or a vector control for 72 (for MDA-MB-231) or 48 hours (for NCI-H460 and Saos-2) by transient transfection were treated with MG132 (20 μM) for 6 hours prior to IB analyses. **E.** IB analyses of MYC and SNAI2 protein levels in Huh7 cells stably expressing FBXO28-mGFP or mGFP and in Hep3b cells stably expressing shFBXO28 or shCtrl for 48 hours.


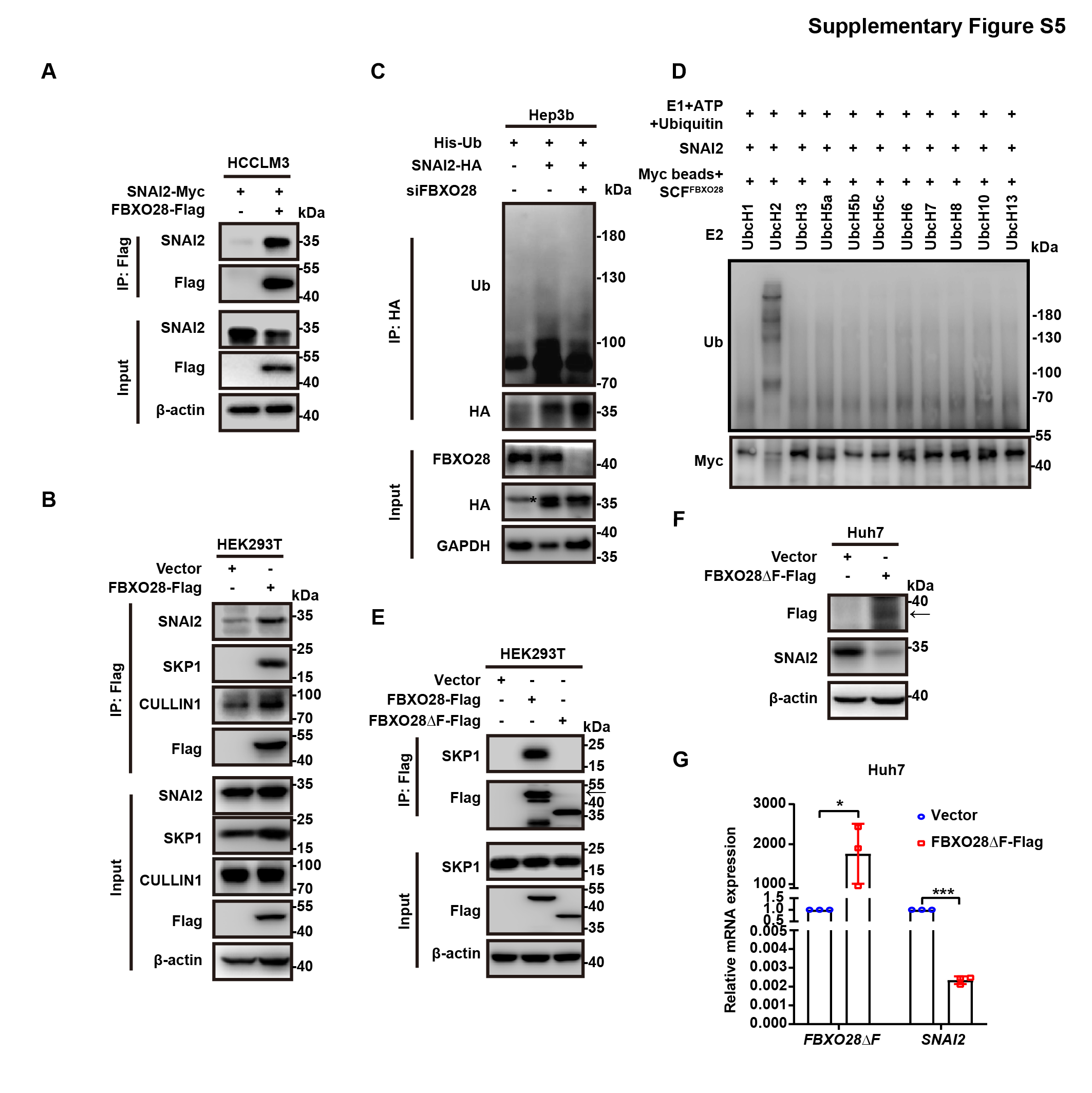


**Supplementary Figure S5.** **A.** HCCLM3 were transfected with pcDNA3.1-SNAI2-Myc (SNAI2-Myc) and pCMV6-FBXO28-Myc-Flag (FBXO28-Flag) or a control vector for 72 hours, and subjected to IP and IB analyses. **B.** HEK293T cells expressing FBXO28-Flag or a vector control for 48 hours by transient transfection were subjected to IP and IB analyses. **C.** Hep3b cells expressing si-Scramble or si-FBXO28 along with SNAI2-HA and His-Ub by transient co-transfection for 72 hours, followed by IP and IB analyses**.** Asterisk indicates non-specific band**. D.** *In vitro* ubiquitination assay using cell immunocomplex pulled down with anti-Myc beads from HEK293T cell extracts after transfection with FBXO28-Flag for 48 hours. The polyubiquitination was detected by IB with anti-Ub antibody. **E.** HEK293T cells expressing FBXO28-Flag or a vector control for 48 hours by transient transfection were subjected to IP and IB analyses. Arrow indicates target band. **F, G.** Huh7 cells expressing FBXO28ΔF-Flag or a control vector by transient transfection for 48 hours were subjected to IB (F) and qRT-PCR (G) analyses. The arrow indicates target band. Data in (G) were presented as mean ± SD from three independent experiments. ^*^*P* < 0.05 and ^***^*P* < 0.001 (independent T test).


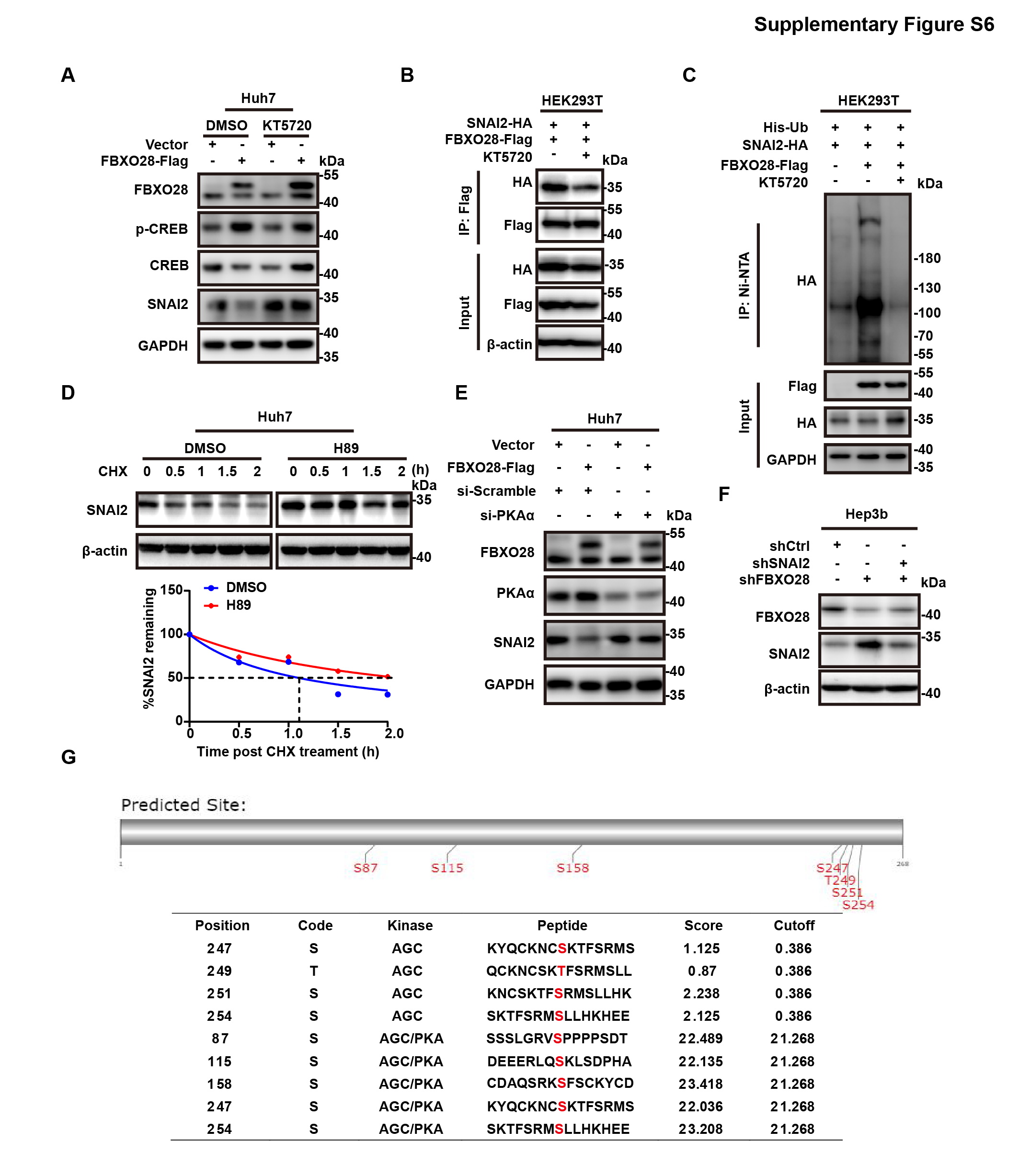


**Supplementary Figure S6. A.** Huh7 cells transiently expressing FBXO28-Flag or a control vector for 24 hours were treated with DMSO or 1 μM KT5720 for 24 hours prior to IB analyses. **B.** HEK293T cells were transfected with indicated plasmids for 24 hours, and then treated with DMSO or 1 μM KT5720 for 24 hours, followed by IP and IB analyses. **C**. HEK293T cells were transfected with indicated plasmids for 48 hours prior to treatment with DMSO or 1 μM KT5720 for 24 hours. The polyubiquitylated proteins were purified by Ni-NTA beads and detected with anti-HA antibody. **D.** Huh7 cells were treated with 0.5 μM H89 for 24 hours and then treated with CHX (10 μg/ml) for the indicated time interval, followed by IB analyses. **E.** Huh7 cells were co-transfected with indicated plasmids and siRNAs for 48 hours prior to IB analyses. **F.** Hep3b cells stably expressing shCtrl, shFBXO28 alone or in combination with shSNAI2 were subjected to IB analyses. **G.** Predicted PKA phosphorylation sites for SNAI2 using the GPS5.0-kinase specific phosphorylation site prediction database (http://gps.biocuckoo.cn). Red residue demonstrates predicted phosphorylated site.

**Supplementary Table 1. RNAs target Sequence (5'-3')**

**siFBXO15 GGATAAAGAAGCTGGTTAT**

**siFBXO17 CCTTCTGGATGTGTATGAA**

**siFBXO24 CCCAGTCAGAGACCTTGTT**

**siFBXO28 GACAAGAGGTTACCAAACT**

**siFBXO46 GTGACTTATACCAGCTCAT**

**siFBXW8 GCCTTTCTTTGATATCCAA**

**siFBXL7 GCTGTTACAATATCTCCAA**

**siPRKACA GGGTGATGCTGGTGAAACA**

**shFBXO28 TTATGTCCTACGACGAAATTA**

**sgFBXO28 GAGCTGGCTAATTTCGTCGT (Rev)**

**Supplementary Table 2. Primers sequences of indicated genes**

**Gene** **Forward sequence (5'-3')** **Reverse sequence (5’-3’)**

***FBXO28*** **TCCTCAGCTTTATGTCCTACGA** **TGGGAGTTGTGCTTTAACTTGT**

***FBXO28ΔF*** **AATGGTGCTGGCGTGACTGTTC** **ACCTATGATCTGGGTCTGCTCTAGC**

***SNAI1*** **ACTGCAACAAGGAATACCTCAG** **GCACTGGTACTTCTTGACATCTG**

***SNAI2* TGTGACAAGGAATATGTGAGCC** **TGAGCCCTCAGATTTGACCTG**

***CDH1*** **ATTTTTCCCTCGACACCCGAT** **TCCCAGGCGTAGACCAAGA**

***VIM*** **TGCCGTTGAAGCTGCTAACTA** **CCAGAGGGAGTGAATCCAGATTA**

***GAPDH*** **CATGAGAAGTATGACAACAGCCT** **AGTCCTTCCACGATACCAAAGT**

**Unprocessed immunoblots for indicated Figures panels.**

**
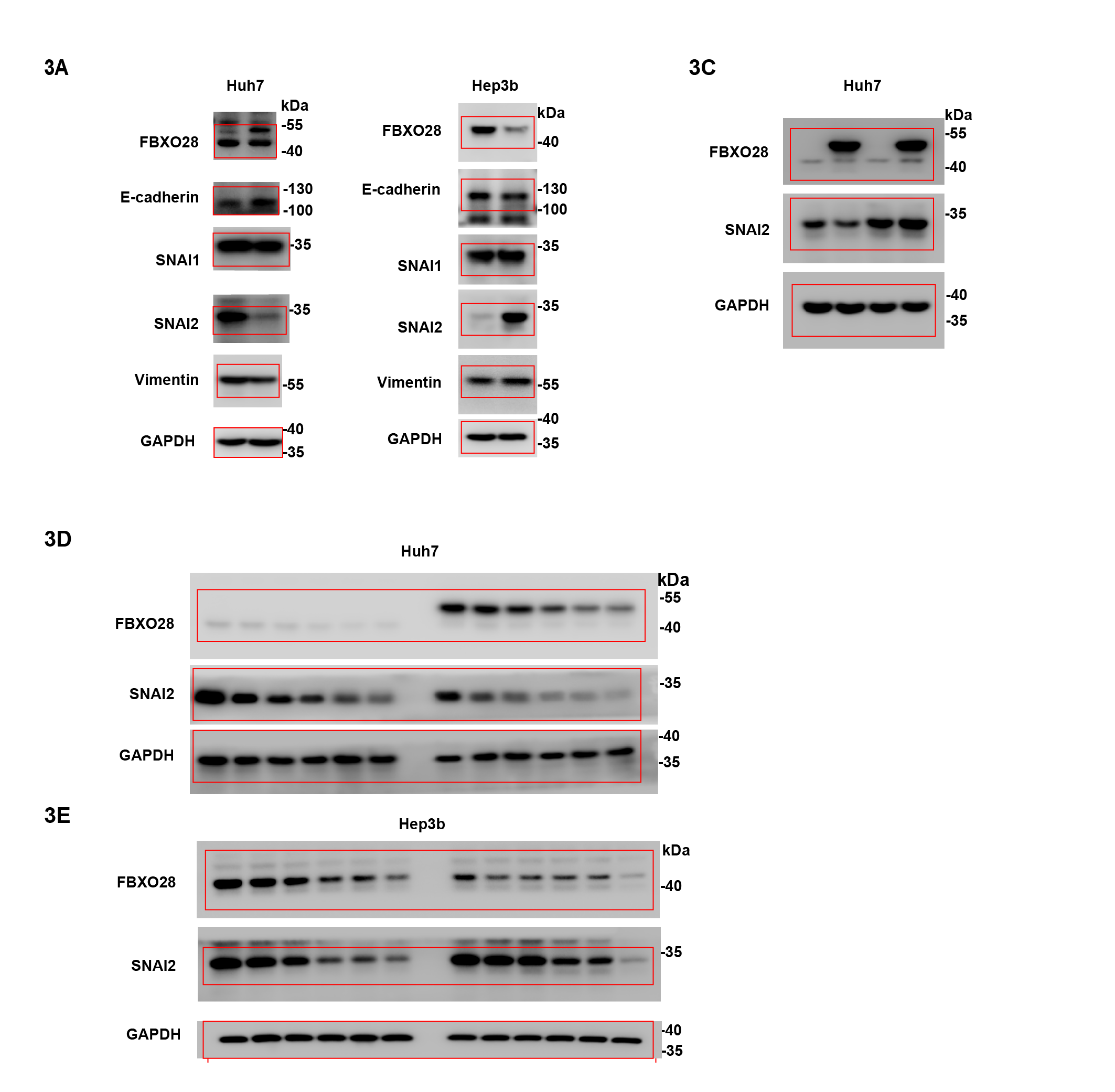
**

**Supplementary Figure S7.** (Unprocessed immunoblots)

**
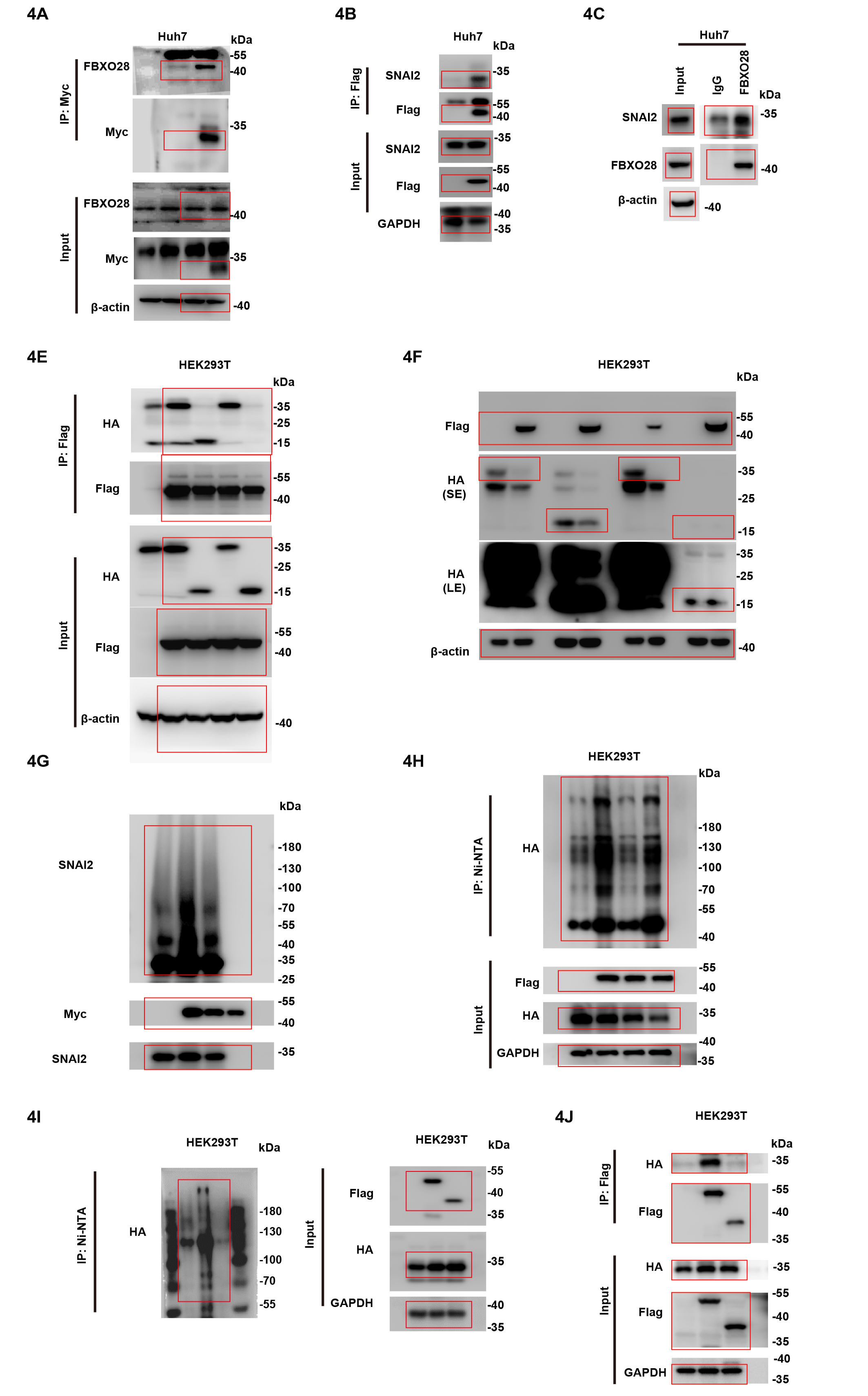
**

**Supplementary Figure S7 (cont’d).** (Unprocessed immunoblots)

**
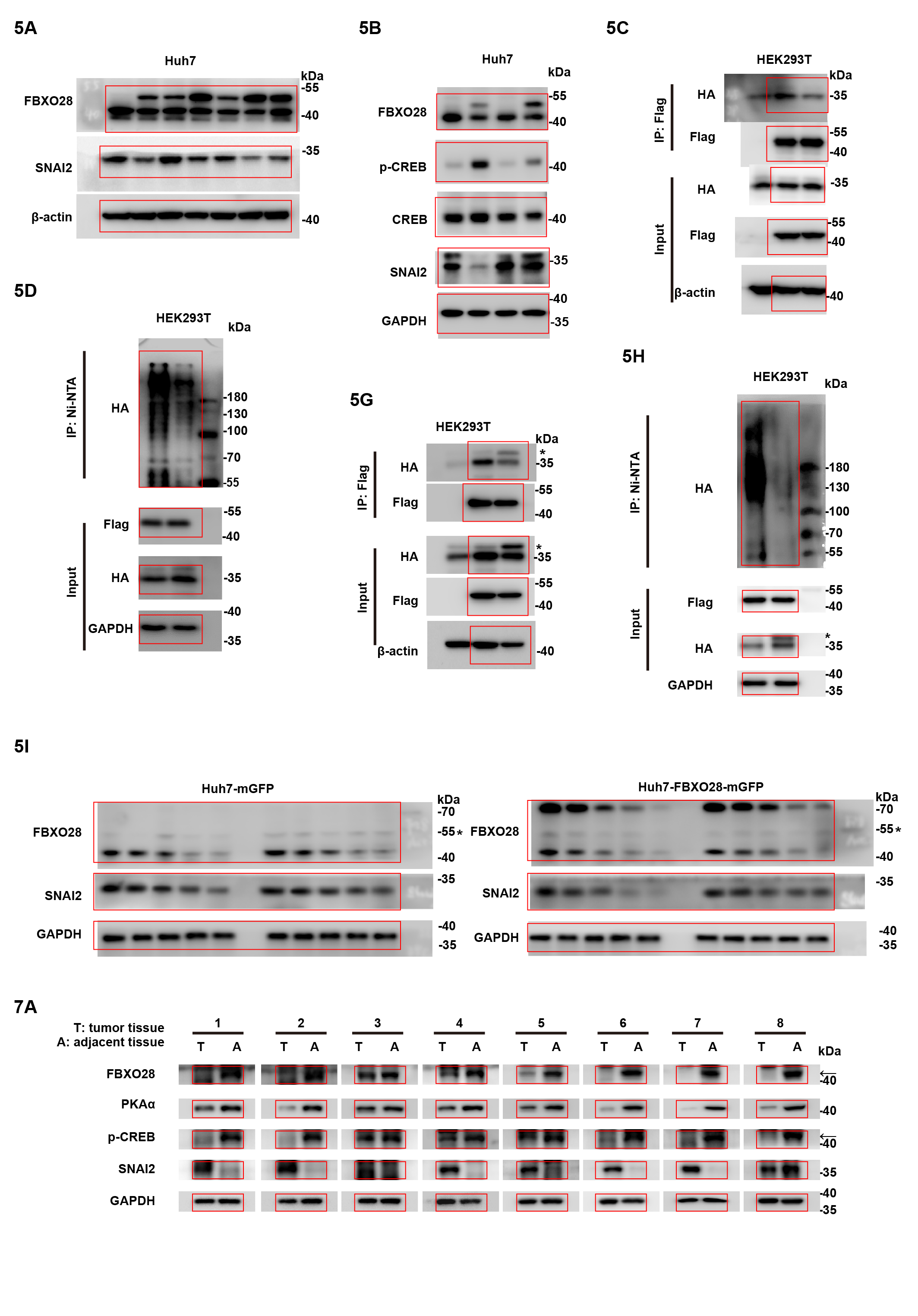
**

**Supplementary Figure S7 (cont’d).** (Unprocessed immunoblots)

**
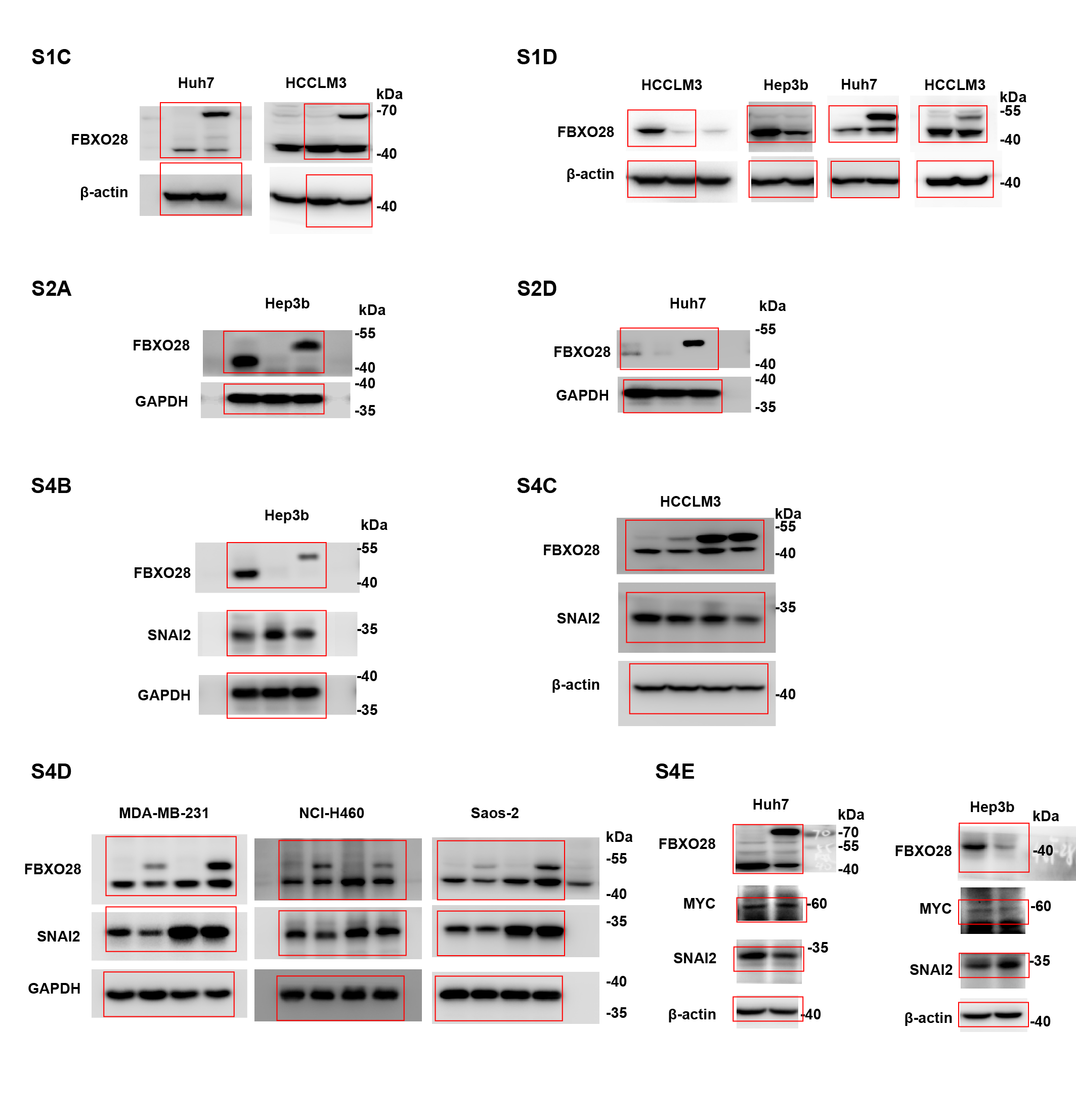
**

**Supplementary Figure S7 (cont’d).** (Unprocessed immunoblots)

**
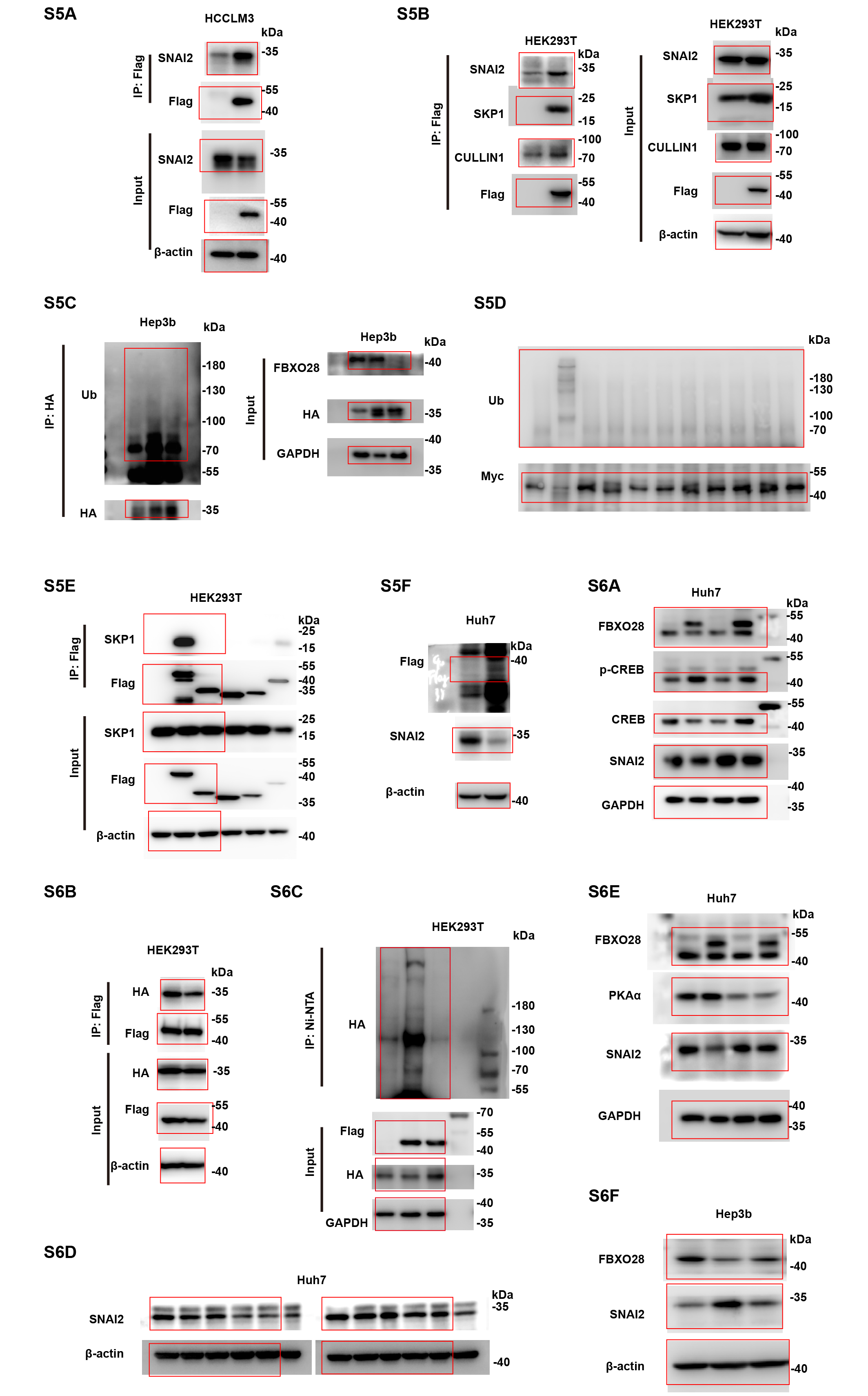
**

**Supplementary Figure S7 (cont’d).** (Unprocessed immunoblots)
